# Confining Li⁺ Solvation in Core–Shell Metal–Organic Frameworks for Stable Lithium Metal Batteries at 100 °C

**DOI:** 10.1007/s40820-025-01988-7

**Published:** 2026-01-05

**Authors:** Minh Hai Nguyen, Jeongmin Shin, Mee-Ree Kim, Quan Van Nguyen, JinHyeok Cha, Sangbaek Park

**Affiliations:** 1 Department of Materials Science and Engineering, Chungnam National University, Daejeon, 34134 Republic of Korea; 2 School of Mechanical Engineering, Chonnam National University, Gwangju, 61186 Republic of Korea

**Keywords:** Quasi-solid-state electrolyte, Metal–organic frameworks, Li metal batteries, Thermal stability, Lithium-ion solvation/de-solvation

## Abstract

The practical deployment of lithium metal batteries remains severely constrained, especially under elevated temperatures. Although metal–organic frameworks (MOFs) improve the thermal stability of liquid electrolytes by capturing them in well-ordered sub-nanopores, interparticle voids between MOF particles readily absorb liquid electrolyte, obscuring our understanding of the intrinsic role of nanopores in directing Li⁺ transport. To address this challenge, we introduce a one-dimensional (1D) MOF model architecture that eliminates interparticle effects and enables direct observation of Li⁺ solvation and de-solvation dynamics. Comparative studies of 1D HKUST-1 and ZIF-8 uncover distinct transport behaviors, supported by both experimental measurements and neural network potential-based molecular dynamics simulations. Building on these insights, we construct a hierarchical core–shell MOF architecture by integrating ZIF-8 (core) and HKUST-1 (shell) onto a hybrid fiber scaffold. This design harnesses the complementary strengths of both MOFs to achieve continuous ion pathways, directional Li⁺ conduction, and improved thermal and electrochemical resilience.

**Supplementary Information:**

The online version contains supplementary material available at 10.1007/s40820-025-01988-7.

## Highlights

- We report the in-situ growth of core–shell metal-organic frameworks on glass fiber, creating a binder-free quasi-solid-state electrolytes (QSSEs) with multiple Li^+^ transport pathways.
- Pore-size-dependent solvation and de-solvation structures of Li^+^ are confined within HKUST-1 and ZIF-8 channels, enabling tailored ion dynamics.
- The core–shell QSSE achieves high Li^+^ conductivity, suppressed dendrite growth, and stable Li plating/stripping under high-temperature conditions.
- Lithium metal batteries with the core–shell QSSE show exceptional cycling stability at 100 °C.

**Supplementary Information:**

The online version contains supplementary material available at 10.1007/s40820-025-01988-7.

## Graphical abstract

**Figure:**
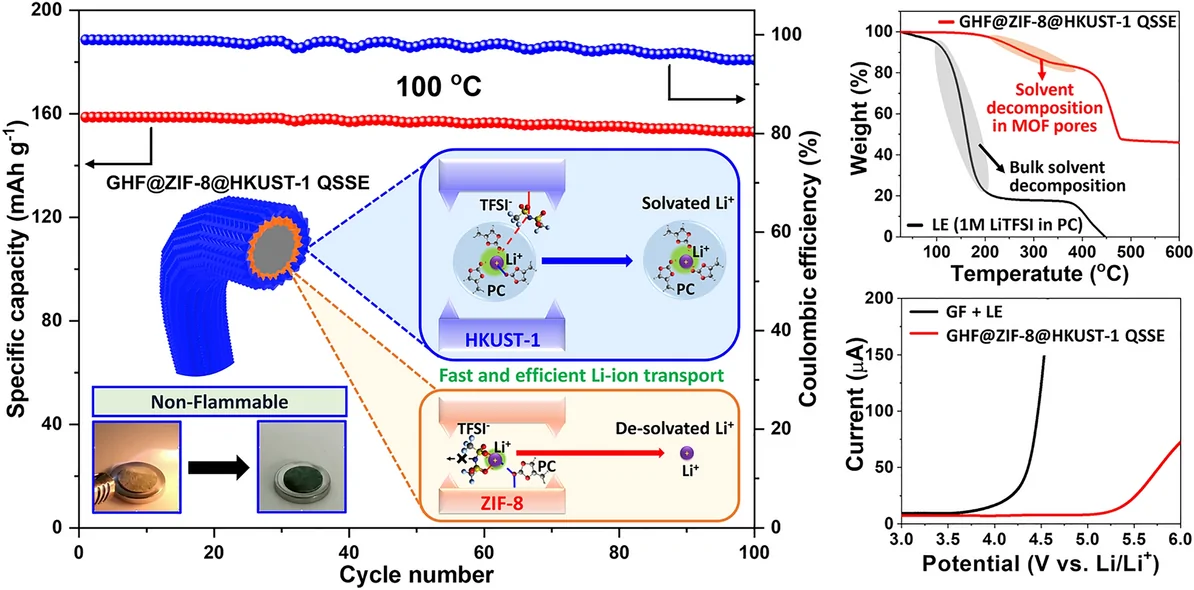

## Introduction

**Fig. 1:**
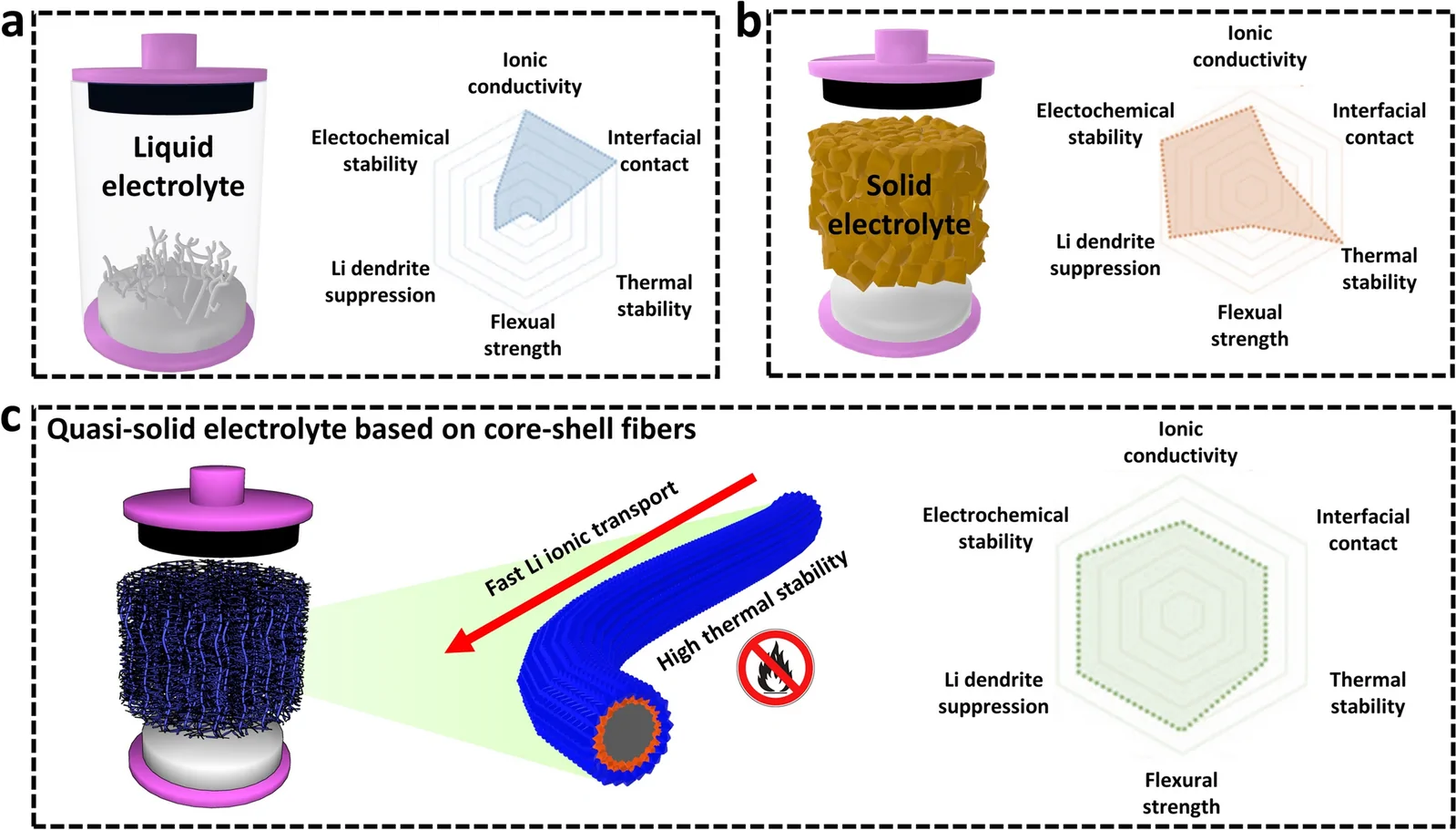
The superior performance of designed QSSE compared to LEs and SSEs. Strengths and limitations of **a** conventional liquid electrolytes and **b** solid-state electrolytes. **c** Exceptional properties of core–shell MOF-based semi-solid electrolytes for stable, high-performance lithium metal batteries

The practical use of high-energy lithium metal batteries (LMBs) is hindered by the inherent instability of traditional liquid electrolytes (LEs) [1, 2], which often causes battery failure and thermal runaway, especially at high temperatures (Fig. 1a) [3–5]. To address this, solid-state electrolytes (SSEs) and quasi-solid-state electrolytes (QSSEs) have emerged [6–8]. SSEs offer thermal/electrochemical stability, a wide electrochemical window, and dendrite suppression [9]. However, their adoption is limited by several intrinsic drawbacks, including low ionic conductivity, poor interfacial compatibility, mechanical brittleness, and manufacturing challenges (Fig. 1b) [10, 11]. As an alternative, QSSEs, incorporating a small amount of LE within a solid matrix [12], bridge the gap between LEs and SSEs, offering enhanced conductivity, interfacial kinetics, and safety (Fig. 1c).

Metal–organic frameworks (MOFs) have emerged as promising hosts due to their well-defined porosity, tunable functionality, and potential to facilitate and regulate lithium-ion transport [13–15]. It enables a pronounced nanoconfinement effect, where Li⁺ ions are selectively guided through sub-nanometer MOF channels, leading to reduced solvent coordination, suppressed ion–solvent clustering, and enhanced directional ion mobility under spatial confinement [16, 17]. Prior studies suggested the nanoconfining mechanism based on free-standing MOF-based separators by binding MOF particles together with binders and coating them directly onto traditional separator materials [18–21], or integrating MOF particles with other materials to form composite matrices, such as polymers, carbon, or SSE particles [22–25]. However, the presence of inter-particle voids absorbed excess liquid electrolyte and obscured the role of intrinsic MOF channels in guiding Li⁺ migration (Fig. S1), hindering clear scientific proof of nanoconfinement effect in MOFs.

In this study, we introduce a one-dimensional (1D) MOF model architecture to isolate and rigorously understand ion transport through MOF nanopores. By directly growing HKUST-1 and ZIF-8 MOFs along individual hybrid fibers, we eliminate the influence of particle–particle interfaces (Fig. S1). This model system reveals direct evidences, for the first time, how Li⁺ solvation structures are governed by pore geometry and framework chemistry, as confirmed through systematic experimental analyses and neural network potential (NNP)-based atomistic simulations. Building on this mechanistic insight, we designed a hierarchical core–shell MOF structure that integrates ZIF-8 core and HKUST-1 shell (Fig. 1c). This architecture merges the solvation-stabilizing capacity of HKUST-1 with the de-solvation-promoting confinement of ZIF-8, creating a seamless and synergistic ion-conducting network. The resultant QSSE achieves stable lithium transport under harsh thermal and electrochemical conditions, overcoming the long-standing limitations of previous MOF-based systems. By resolving the fundamental challenges in ion transport, solvation regulation, and structural integration, our work establishes a new paradigm for MOF-based QSSEs, offering a clear pathway toward safe, high-performance lithium metal batteries capable of reliable operation even under extreme environments.

## Experimental Section

### Materials

Glass fiber membranes (GF, Grade GF/A) were purchased from WHATMAN and used as substrates for the in situ growth of metal–organic frameworks (MOFs). The precursor materials for MOF synthesis, including copper (II) nitrate trihydrate (Cu(NO₃)₂·3H₂O, 99%), lauric acid (99%), 1,3,5-benzenetricarboxylic acid (95%), zinc nitrate hexahydrate (Zn(NO₃)₂·6H₂O, 98%), and 2-methylimidazole (2-MIM, 99%), were obtained from Sigma-Aldrich. Chemicals used for surface modification of the glass fibers included sodium hydroxide (NaOH, 98%) from SAMCHUN, as well as (3-aminopropyl)triethoxysilane (APTES, 99%) and glutaraldehyde (25% in H₂O), both purchased from Sigma-Aldrich. Bovine serum albumin (98.5%) was used as a biological component to prepare GF-based hybrid fibers and was also sourced from Sigma-Aldrich. Lithium bis(trifluoromethanesulfonyl)imide (LiTFSI, 99.9%) was purchased from Sigma-Aldrich and used as the lithium salt. Materials for cathode preparation, including LiFePO₄ powder and polyvinylidene fluoride (PVDF, 99.5%), were obtained from MTI Corporation, while Super P conductive carbon was supplied by TIMCAL. Organic solvents, including 1-butanol (99.9%) and propylene carbonate (PC, 99.7%), were purchased from Sigma-Aldrich; ethanol (99.9%) was obtained from Thermo Fisher Scientific, and N-methyl-2-pyrrolidone (NMP, 99.5%) from SAMCHUN. Lithium metal chips were purchased from NEBA Lithium Solution. CR2032 coin cell components were supplied by Sunny Tech Co., Ltd., and pouch cell components were acquired from SINOPRO.

### Sample Preparation

#### Synthesis of ZIF-8 on GHF

GHF@ZIF-8 membranes were prepared by combining commercial glass fiber (GF) with bovine serum albumin (BSA) to form a GF-based hybrid fiber (GHF). First, the GF membranes underwent surface hydrolysis by immersion in a 1 M NaOH solution for 1 h. After thoroughly rinsing with deionized (DI) water, the membranes were dried at 60 °C for 10 h. The membranes were then immersed in a 5% APTES solution in acetone for 12 h, followed by rinsing with acetone and drying at 60 °C for another 10 h. Subsequently, the membranes were soaked in a 5% glutaraldehyde (GA) solution in deionized (DI) water for 6 h, washed with DI water, and dried at 60 °C for 2 h. Finally, the membranes were incubated in a 1% BSA solution (in DI water) for 24 h. After rinsing and drying at 60 °C for 10 h, the resulting GHF membranes were ready for MOF growth. For in situ ZIF-8 growth, the GHF membranes were vertically immersed in three separate vials, each containing 20 mL of zinc nitrate hexahydrate (Zn(NO₃)₂·6H₂O) solutions at concentrations of 0.112, 0.243, and 0.73 g, respectively, and soaked for 10 min. Then, 20 mL of 2-methylimidazole (2-MIM) linker solutions containing 2.29, 1.00, and 1.99 g of 2-MIM, respectively, was slowly added to the corresponding vials. The mixtures were left undisturbed at room temperature for 24 h to allow ZIF-8 crystals to grow on the GHF membranes, forming distinct morphologies depending on the precursor concentrations. Afterward, the membranes were washed with DI water and ethanol and then dried in a vacuum oven at 180 °C for 12 h. The final GHF@ZIF-8 membranes were stored in an argon-filled glove box for further experiments.

#### Synthesis of HKUST-1 on GHF

Following a similar procedure to that of GHF@ZIF-8, the prepared GHF membranes were also used as substrates for the in situ growth of HKUST-1. To prepare the salt solutions, copper (II) nitrate trihydrate (0.68 mmol) was mixed with varying amounts of lauric acid (18.90, 38.00, and 57.04 mmol) and dissolved in 50 mL of 1-butanol. The linker solution was prepared separately by dissolving 1,3,5-benzenetricarboxylic acid (0.38 mmol) in 50 mL of 1-butanol. The GHF membranes were vertically immersed in Teflon-lined stainless-steel autoclaves containing the salt solutions and allowed to stand for 1 h. Subsequently, the linker solution was slowly added to each autoclave. After sealing, the autoclaves were heated at 140 °C for 2 h to facilitate the growth of HKUST-1 crystals with distinct morphologies on the GHF substrates. The resulting membranes were thoroughly washed with 1-butanol and ethanol, dried in a vacuum oven at 180 °C for 12 h, and stored in an argon-filled glove box for subsequent experiments.

#### Synthesis of MOF Core–Shells on GHF

The synthesis of MOF core–shell structures on GHF followed a similar procedure to that used for preparing GHF@HKUST-1, with the key difference being the use of pre-fabricated GHF@ZIF-8 membranes as the substrate instead of GHF. The GHF@ZIF-8 membranes were vertically immersed in Teflon-lined stainless-steel autoclaves containing a salt solution composed of copper (II) nitrate trihydrate (0.68 mmol) and lauric acid (38 mmol) dissolved in 50 mL of 1-butanol. After soaking for 1 h, a linker solution consisting of 1,3,5-benzenetricarboxylic acid (0.38 mmol) in 50 mL of 1-butanol was slowly added to each autoclave. The sealed autoclaves were then heated at 140 °C for 2 h to induce the growth of an HKUST-1 shell layer on the GHF@ZIF-8 cores, forming a MOF core–shell architecture. After the reaction, the membranes were thoroughly rinsed with 1-butanol and ethanol, dried in a vacuum oven at 180 °C for 12 h, and stored in an argon-filled glove box for subsequent experiments.

#### Preparation of Quasi-Solid Electrolytes

To prepare the liquid electrolyte, 2.88 g of LiTFSI was thoroughly dissolved in 10 mL of propylene carbonate (PC) to obtain a 1 M LiTFSI solution. For the preparation of quasi-solid electrolytes, the MOF-based membranes underwent a two-step activation process. First, the GHF@MOF separators were thermally activated by immersing them in the liquid electrolyte and heating at 60 °C for 24 h. This step is referred to as thermal activation. Next, electrochemical activation was performed by assembling the thermally activated membranes into symmetric Li//GHF@MOF//Li cells with 1 M LiTFSI liquid electrolyte and cycling them at a current density of 0.5 mA cm⁻^2^ for 10 cycles. After activation, the quasi-solid-state MOF-based electrolytes were obtained by disassembling the cells, gently rinsing the GHF@MOF membranes with PC to remove excess liquid electrolyte, and drying them in a vacuum oven at 60 °C for 24 h. The final quasi-solid electrolytes were stored in an argon-filled glove box and later used for battery assembly in this study. The entire two-step activation process was conducted inside an argon-filled glove box to prevent moisture and oxygen interference.

#### Preparation of LFP Cathode

LiFePO₄ powder, acetylene black (carbon powder), and polyvinylidene fluoride (PVDF) were mixed in a mass ratio of 8:1:1 using N-methyl-2-pyrrolidone (NMP) as the solvent. The resulting slurry was homogenized using a ball mill and then cast onto carbon-coated aluminum foil using the blade-casting method. The coated electrodes were dried under a vacuum at 110 °C for 12 h. The areal loading of active material was approximately 3.2 mg cm⁻^2^. After drying, the LFP cathodes were punched into circular disks with a diameter of 14 mm for coin cell assembly and stored in an argon-filled glove box to prevent moisture exposure.

### Sample Characterizations

The morphology of MOFs grown on GHF membranes was examined using field-emission scanning electron microscopy (FESEM, SU7000, HITACHI) at an accelerating voltage of 10 kV. The core–shell structure of the MOFs was further analyzed by cross-sectional SEM imaging of the fibers, and the corresponding elemental distribution was investigated using energy-dispersive X-ray spectroscopy (EDS, Ultim Max65, Oxford). The crystalline structures of GHF@MOF membranes before and after electrochemical activation were characterized by X-ray diffraction (XRD, D8 ADVANCE, Bruker) using Cu Kα radiation (λ = 1.54 Å) at a scan rate of 5° min⁻^1^ over a 2θ range of 5°–40°. To evaluate the thermal stability of the MOF-based quasi-solid-state electrolytes and the standard LiTFSI liquid electrolyte, thermogravimetric analysis (TGA) was performed using a Mettler Toledo thermal analyzer from 25 to 600 °C at a heating rate of 10 °C min⁻^1^. Raman spectroscopy (Model: HEDA, Manufacturer: WEVE) was employed to probe the interaction of liquid electrolyte species inside and outside the MOF pores. Spectra were collected using a 532 nm argon-ion laser and a 1200 grating, with scattered light acquired at a 90° angle relative to the incident beam. To investigate the solvation structure and Li⁺ transport kinetics within the activated MOF-based separators, solid-state ^7^Li magic-angle spinning (MAS) nuclear magnetic resonance (NMR) spectroscopy and T₁ relaxation measurements were conducted. ^7^Li MAS NMR spectra were recorded using a 3.2 mm solid-state probe spinning at 15 kHz on a 600 MHz NMR spectrometer (VARIAN, INOVA). T₁ relaxation times were determined using an inversion recovery pulse sequence with 14–16 delay points and calculated using the equation *I* = *I₀(1 – 2e⁻ᵗ⁄ᵀ*^*1*^*)*, where *I* is the peak intensity at time *t*, *I₀* is the saturation intensity, and *T₁* is the longitudinal (spin–lattice) relaxation time. Attenuated total reflection Fourier transform infrared spectroscopy (ATR-FTIR) was performed using a VERTEX 80v spectrometer (Bruker) equipped with a Platinum Diamond ATR accessory to detect trace amounts of residual LiTFSI liquid electrolyte within MOF pores after activation by monitoring characteristic vibrational bands.

### Cell Assembly and Electrochemical Measurements

To prevent the influence of moisture and oxygen, all CR2032 coin cells were assembled in an argon-filled glove box. For the fabrication of Li//LFP batteries, GHF@MOF-based quasi-solid-state electrolytes (QSSEs) were integrated with LFP cathodes and lithium metal chips in CR2032 coin cells without the addition of liquid electrolyte (LE). For comparison, a reference Li//LFP cell was also assembled using 100 µL of conventional LE (1 M LiTFSI in propylene carbonate) and a commercial glass fiber (GF) separator. All Li//LFP cells were cycled between 2.0 and 4.2 V vs. Li/Li⁺ at a 1 C rate using a computer-controlled battery cycler (Neware) at an elevated temperature of 100 °C. To assemble Li//Li symmetric cells, two lithium chips were used as electrodes, while either the MOF-based QSSEs or commercial GF separator was placed between them. These symmetric cells were cycled at 1 mAh cm⁻^2^ (1 mA cm⁻^2^ for 1 h) at room temperature using the same battery tester. Electrochemical impedance spectroscopy (EIS) measurements were taken using an impedance spectrometer (Solartron SI 1260, AMETEK SI) over a frequency range from 1 Hz to 10 MHz at various temperatures from 25 to 70 °C. Linear sweep voltammetry (LSV) was conducted using a potentiostat/galvanostat (IVIUM Technologies, Eindhoven, Netherlands) over a voltage window from 3 to 6 V to evaluate the electrochemical stability window. The lithium-ion transference number (*tₗᵢ⁺*) was determined in Li//Li symmetric cells incorporating either the prepared QSSEs or commercial GF soaked with LE (1 M LiTFSI in PC) using a DC polarization method. A constant voltage of 10 mV was applied, and EIS measurements were taken both before and after polarization using the same potentiostat. The *tₗᵢ⁺* value was calculated based on Bruce’s equation: *tₗᵢ⁺* = *Iₛ (ΔV – I₀R₀) / [I₀ (ΔV – IₛRₛ)]*, where ΔV is the applied polarization voltage (10 mV), *I₀* is the initial current, *Iₛ* is the steady-state current, *R₀* is the initial interfacial resistance, and *Rₛ* is the steady-state resistance.

### Simulation Detail

#### Density Functional Theory Simulation

All density functional theory (DFT) calculations were carried out using the CASTEP module in Materials Studio 2024 (BIOVIA, San Diego, CA, USA) [26]. Ultrasoft pseudopotentials were generated on the fly, and a plane-wave cutoff energy of 650 eV was employed [27]. Exchange–correlation interactions were treated using the Perdew–Burke–Ernzerhof (PBE) functional within the generalized gradient approximation (GGA) [28, 29].

Geometry optimizations were performed with convergence thresholds of 2.0 × 10⁻^5^ eV atom^−1^ for energy, 0.05 eV Å^−1^ for force, 0.1 GPa for stress, and 0.002 Å for atomic displacement. Due to the large unit cell sizes of the systems, all periodic DFT calculations were conducted using a Γ-point (1 × 1 × 1 Monkhorst–Pack grid). The unit cells of HKUST-1 and ZIF-8 were modeled as cubic boxes with lattice parameters of 19.0 and 17.0 Å, respectively.

The binding energy of Li⁺ coordinated with the MOF-TFSI^−^-PC system was evaluated as defined below:

1 $$E_{{\text{Binding Energy}}} = E_{{{\mathrm{Li}}^{ + } @{\mathrm{MOF}} + {\mathrm{TFSI}} + {\mathrm{PC}}}} {-}\left( {E_{{{\mathrm{Li}}^{ + } + }} + E_{{{\mathrm{MOF}} + {\mathrm{TFSI}} + {\mathrm{PC}}}} } \right)$$

where *E*_Li⁺@MOF+TFSI+PC_ is the total energy of the MOF system containing Li⁺, *E*_Li⁺_ is the energy of the isolated Li⁺ ion, and *E*_MOF+TFSI+PC_ is the total energy of the MOF and coordinating species (TFSI^−^ and PC) in the absence of Li^+^. These DFT-calculated energies were used to evaluate the relative binding strength of Li^+^ in different environments and provided the initial structural and energetic basis for subsequent molecular dynamics simulations.

#### Molecular Dynamics Simulation

Molecular dynamics (MD) simulations were performed with the universal neural network potential (UNNP), a machine learning-based interatomic model trained on extensive first-principles datasets. The UNNP was developed from DFT calculations covering 96 elements and diverse structural environments, enabling accurate prediction of atomic energies and forces across a broad range of configurations [30]. To construct confined Li-ion systems, one Li⁺ ion, one TFSI⁻ anion (15 atoms), and twelve PC molecules (144 atoms) were placed within a single-unit pore of either HKUST-1 or ZIF-8, corresponding to a 1:12 LiTFSI:PC ratio. This model was subsequently expanded to a 2 × 2 × 2 supercell, resulting in final system sizes of 2624 and 3584 atoms, respectively.

Each supercell thus contained 8 Li⁺ ions, 8 TFSI⁻ anions, and 96 PC molecules, with periodic boundary conditions applied in all directions. The final simulation box dimensions were approximately 3.8 × 3.8 × 3.8 nm^3^ for HKUST-1 and 4.32 × 4.32 × 4.32 nm^3^ for ZIF-8. The pore volumes were approximately 1.15 nm^3^ per pore for HKUST-1 and 0.63 nm^3^ per pore for ZIF-8, consistent with literature-reported accessible volumes [31, 32]. The thickness of the MOF walls corresponds to the metal cluster separation in the respective unit cells, approximately 7.2 Å for HKUST-1 and 5.6 Å for ZIF-8.

Following geometry optimization via LBFGS (fmax = 0.005 eV Å^−1^, 1000 steps), simulations were carried out in the NVT ensemble at 300 K using a Langevin thermostat (friction coefficient = 0.002 fs⁻^1^). A time step of 0.1 fs was used, with total simulation durations of 500 ps. Atomic velocities were initialized using a Maxwell–Boltzmann distribution, and trajectories were recorded at 1 ps intervals. Diffusivities of Li⁺ and TFSI⁻ ions were calculated by fitting the linear region of the MSD curves in the 200–400 ps window.

## Results and Discussion

### Fabrication of Single MOFs Grown on GHF and Confinement of LE in their Pores

**Fig. 2:**
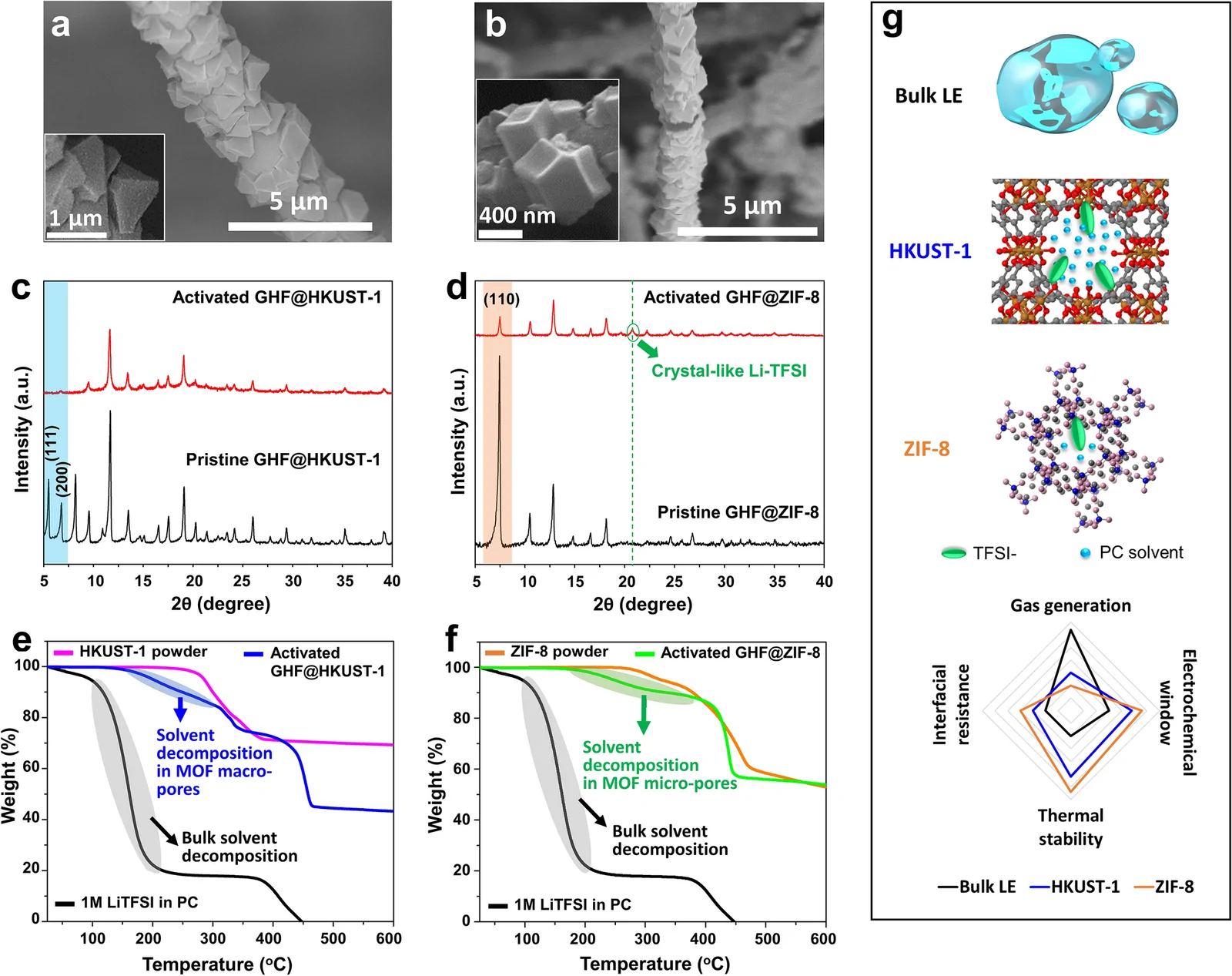
Morphologies of single MOFs grown on GHF and liquid electrolyte infiltration. SEM images of as-synthesized **a** truncated octahedral HKUST-1 and **b** rhombic dodecahedral ZIF-8 particles grown on GHF. XRD patterns of pristine and activated **c** GHF@HKUST-1 and **d** GHF@ZIF-8. Thermogravimetric analysis (TGA) curves of activated **e** GHF@HKUST-1 and **f** GHF@ZIF-8 separators in comparison with the typical liquid electrolyte and corresponding MOF powders. While the conventional LE (1 M LiTFSI in PC) showed poor thermal stability (highlighted in gray), the activated MOF-based QSSEs exhibited significantly enhanced thermal stability. The activated GHF@ZIF-8 revealed a slower decomposition rate over a broader temperature range (marked in green) compared to activated GHF@HKUST-1 (marked in blue). **g** Schematic depiction of the limitations of conventional liquid electrolytes and the features of MOF-based semi-solid electrolytes with varying pore sizes

For a systematic investigation of the dependence of solvation structures and Li^+^ ion transport kinetics on MOF pore size, we deposited ZIF-8 and HKUST-1, with different pore size distributions, on glass-based hybrid fiber (GHF) membranes (Fig. 2a, b) after optimizing structural/electrochemical stability (Figs. S2 and S3; see Notes S1 and S2 for the detailed discussion). After the infiltration of LE into the MOF pores, the XRD peak intensities of both MOFs decreased, particularly at lower 2θ angles such as (110) for ZIF-8 and (111) and (200) for HKUST-1 (Fig. 2c, d). This intensity reduction indicates that electrolyte molecules were confined within the MOF pores, particularly within the crystallographic planes contributing to these characteristic peaks. Notably, a new XRD peak at 20.7° emerged in activated GHF@ZIF-8, corresponding to the formation of crystal-like Li-TFSI, which was not observed in GHF@HKUST-1. It suggests distinct interactions between Li^+^ cations, TFSI^−^ anions, and PC molecules within different MOF pore environments. Remarkably, GHF@ZIF-8 and GHF@HKUST-1 contained only 0.96 and 1.02 mg cm⁻^2^ of electrolyte, far less than typical LE amount (~ 60 mg cm⁻^2^) in LMBs (Table S1; see Note S3 for the detailed discussion). Thermogravimetric analysis (TGA) showed superior thermal stability of MOF-based QSSEs: While conventional LE (1 M LiTFSI in PC) showed rapid solvent decomposition occurring above 100 °C, the activated MOF-based QSSEs withstood temperatures exceeding 200 °C (Fig. 2e, f). Specifically, the weight loss profile of activated GHF@ZIF-8 revealed a slower decomposition rate over 200–400 °C compared to activated GHF@HKUST-1 (180–300 °C). This is attributed to the sub-nanoconfinement effect, which arises from the restriction of a minimal amount of LE within the nanoporous MOF hosts. Unlike MOF thin-film-based QSSEs [33], where rapid weight loss at lower temperatures was observed due to LE residing at particle boundaries and surface interfaces, our 1D MOFs successfully eliminate interfacial electrolyte accumulation (Fig. S1), improving thermal stability. Based on these findings, we summarize the characteristics of our MOF-based QSSEs in comparison with conventional LE, as illustrated in Fig. 2g (Fig. S4; see Note S4 for the detailed discussion).

### Solvation Structures and Ion Transport Properties in GHF@MOF QSSEs

**Fig. 3:**
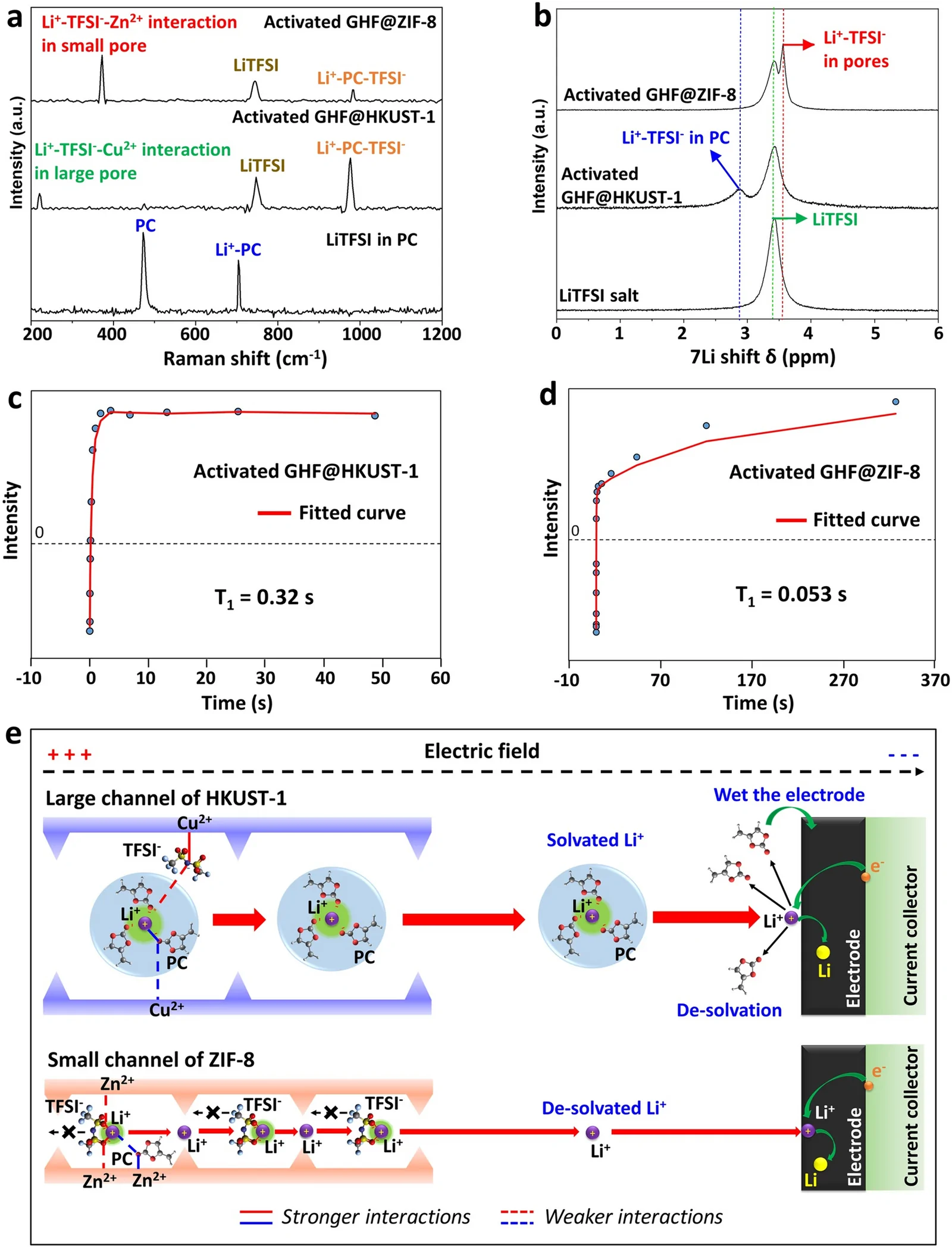
Interactions and ionic transport pathways in pores of single MOFs. **a** Raman spectra of LiTFSI in PC, GHF@HKUST-1, and GHF@ZIF-8 QSSEs. **b** ^7^Li Solid-state MAS NMR spectra of pure LiTFSI salt, activated GHF@HKUST-1, and GHF@ZIF-8 QSSEs. Inversion recovery plots, fitted curves, and calculated T1 of **c** GHF@HKUST-1 and **d** GHF@ZIF-8 QSSEs. **e** The proposed Li^+^ transportation in different MOF channels

Raman spectra (Fig. 3a) showed solvated Li⁺ coordination in HKUST-1 and de-solvated Li⁺ coordination in ZIF-8 due to differences in pore size and nanoconfinement effects, which influence ion–solvent–MOF interactions (see Note S5 for the detailed discussion). Solid-state NMR further confirmed that nanoscale confinement in MOFs plays a decisive role in tuning the solvation structure and ion transport behavior (Fig. 3b–d). The ^7^Li MAS NMR spectrum of LiTFSI salt exhibited a single broad peak, corresponding to strong Li^+^-TFSI^−^ interactions within the crystalline structure of pure LiTFSI salt. Interestingly, the spectra of LiTFSI-based LE confined within different MOF pores revealed distinct Li^+^ environments beyond the unactivated LiTFSI salt within the GHF@MOF membrane body. The NMR spectrum of activated GHF@HKUST-1 exhibited a chemical shift toward higher frequencies, whereas a lower-frequency peak was observed in activated GHF@ZIF-8 (Fig. 3b), corresponding to Li^+^-TFSI^−^-PC interactions in large pores (solvated Li^+^ structure) and Li^+^-TFSI^−^ interactions in small pores (de-solvated Li^+^ structure), respectively. This observation aligns well with the Raman results (Fig. 3a). This clearly highlights the distinct chemical environments of Li⁺ in 1D HKUST-1 and ZIF-8, more sharply resolved than in our previous studies using MOF thin-film QSSEs [33].

In addition, Li^+^ mobility within the MOF frameworks was investigated using spin–lattice relaxation time (T1) measurements in ^7^Li solid-state NMR (Fig. 3c, d). A shorter T1 time corresponds to higher species mobility [34]. The QSSEs based on GHF@MOF exhibited high Li^+^ mobility, with values ranging from 0.053 to 0.32 s, far shorter than those reported for pure LiTFSI salt in previous studies (typically on the order of tens of seconds) [33]. Notably, the T1 value of Li^+^ within the ionic channels of ZIF-8 (0.053 s) was significantly shorter than that in HKUST-1 (0.32 s). This difference can be attributed to the de-solvated Li^+^ structure and the restricted mobility of large TFSI^−^ anions within the small ZIF-8 channels, allowing Li^+^ ions to migrate freely under an applied electric field without significant hindrance (illustrated in Fig. 3e). In contrast, the solvation structure of Li^+^ surrounded by multiple PC molecules in HKUST-1 channels led to reduced mobility. Furthermore, de-solvated Li^+^ exiting the ZIF-8 channels can directly interact with electrons at the electrode surface to form Li more efficiently, whereas solvated Li^+^ leaving the larger HKUST-1 channels must undergo a de-solvation process to remove the PC solvation shell at the electrode surface before electron interaction. Consequently, the activation energy of QSSEs based on GHF@ZIF-8 is lower than that of GHF@HKUST-1 QSSEs (Fig. S5; see Note S6 for the detailed discussion).

### Demonstration of Solvation Structures of Li^+^ and Li-ion Transport in Single MOFs by DFT and MD Simulations

**Fig. 4:**
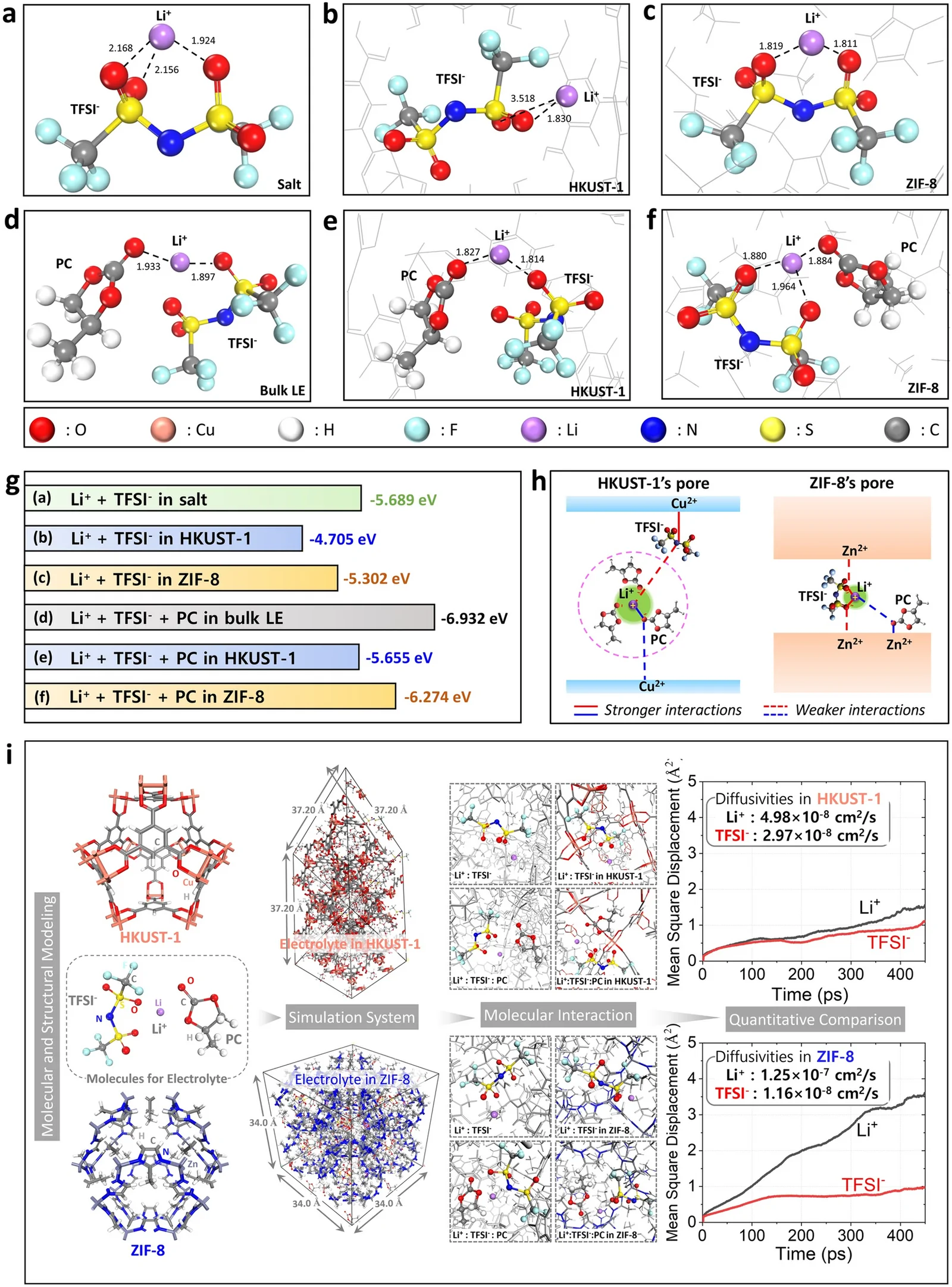
DFT and MD simulations of Li@MOF systems. Simulation snapshots of PC, TFSI⁻, and Li^+^ ions (purple spheres) in **a** bulk liquid electrolyte (LE) system and **b** the pore of HKUST-1 and **c** ZIF-8. Simulation snapshots of TFSI⁻ and Li^+^ ions in **d** bulk LiTFSI salt system and the pore of **e** HKUST-1 and **f** ZIF-8. **g** Summary of binding energy of Li^+^ ion in various systems. **h** Proposed Li^+^ solvation structure in large pore of HKUST-1 and Li^+^ de-solvation structure in small pore of ZIF-8. **i** Neural network potential (NNP)-based molecular dynamics (MD) simulation process: Molecular and structural modeling → Simulation system modeling → Investigation of molecular interaction → Calculation of mean square displacement (MSD) of Li^+^ and TFSI.^−^ ions in HKUST-1 and ZIF-8 as a function of the simulation time (quantitative comparison)

To elucidate the solvation structures of Li⁺ ions within different MOF nanochannels, we calculated the binding energies of relevant species both inside and outside the MOF pores using density functional theory (Fig. 4a–f). When these species were confined within MOF pores, their binding energies significantly decreased (Fig. 4g), indicating that MOFs effectively weaken Li^+^-TFSI⁻ and Li^+^-PC interactions, thereby facilitating Li^+^ transport within the pores compared to bulk conditions. Li^+^ ions interact more strongly with TFSI⁻ within the confined space of ZIF-8, leading to the formation of a de-solvated, crystal-like Li-TFSI structure, as discussed in Fig. 2c, whereas simulations of individual PC solvent molecules within MOF pores revealed stronger PC-MOF interactions in ZIF-8 than in HKUST-1 (Fig. S6). This not only facilitates Li^+^ de-solvation within the small pores of ZIF-8 but also prevents solvent leakage from the porous structure. As a result, ZIF-8-based QSSEs exhibit improved thermal stability but reduced electrode wettability. Conversely, QSSEs based on HKUST-1, with a predominantly solvated Li^+^ structure in its large pores and weaker PC-MOF interactions, offer better electrode wettability, thereby reducing electrolyte/electrode interfacial resistance at the cost of lower thermal stability. The detailed solvation and de-solvation structures of Li^+^ in different MOF pore architectures are illustrated in Fig. 4h (Fig. S6; see Note S7 for the detailed discussion).

To complement our DFT-based analysis, we further performed molecular dynamics (MD) simulations built upon a deep learning architecture with the preferred potential (PFP), a universal neural network potential (UNNP) trained on an extensive DFT dataset covering 96 elemental combinations (see Note S8 for the detailed discussion). Li⁺ exhibited much higher mobility in ZIF-8 (1.25 × 10^–7^ cm^2^ s^−1^) than in HKUST-1 (4.98 × 10^–8^ cm^2^ s^−1^) (Fig. 4i), which is consistent with ^7^Li NMR measurements (Fig. 3c, d). In contrast, TFSI⁻ anions exhibited significantly lower diffusivity in both systems: 1.16 × 10⁻^8^ cm^2^ s⁻^1^ in ZIF-8 and 2.97 × 10⁻^8^ cm^2^ s⁻^1^ in HKUST-1. The mean square displacement (MSD) curves of TFSI⁻ were nearly flat in both cases, confirming severe restriction of anion mobility. This immobilization stems from distinct mechanisms: ZIF-8 physically restricts TFSI⁻ via sub-nanometer pore apertures, while HKUST-1 suppresses TFSI⁻ motion through strong coordination with Cu^2^^+^ open metal sites. As a result, lithium-ion transference numbers (${t}_{{Li}^{+}}$), measured experimentally, show significant enhancement in both systems compared to bulk LE (${t}_{{Li}^{+}}$ = 0.154), reaching 0.36 in GHF@HKUST-1 and a notably higher value of 0.57 in GHF@ZIF-8 (Fig. S7). These results demonstrate that the selective transport properties of GHF@MOF-based QSSEs are determined by the combined influence of MOF pore geometry, metal–ion coordination chemistry, and solvation structure. By strategically tuning these factors, it is possible to design electrolyte architectures that offer fast lithium-ion mobility, suppressed anion transport, high transference numbers, and enhanced stability, which are key attributes for enabling the next generation of high-energy and safe lithium metal batteries.

### Design of Core–Shell MOF Grown on GHF for a High-Performance QSSE

**Fig. 5:**
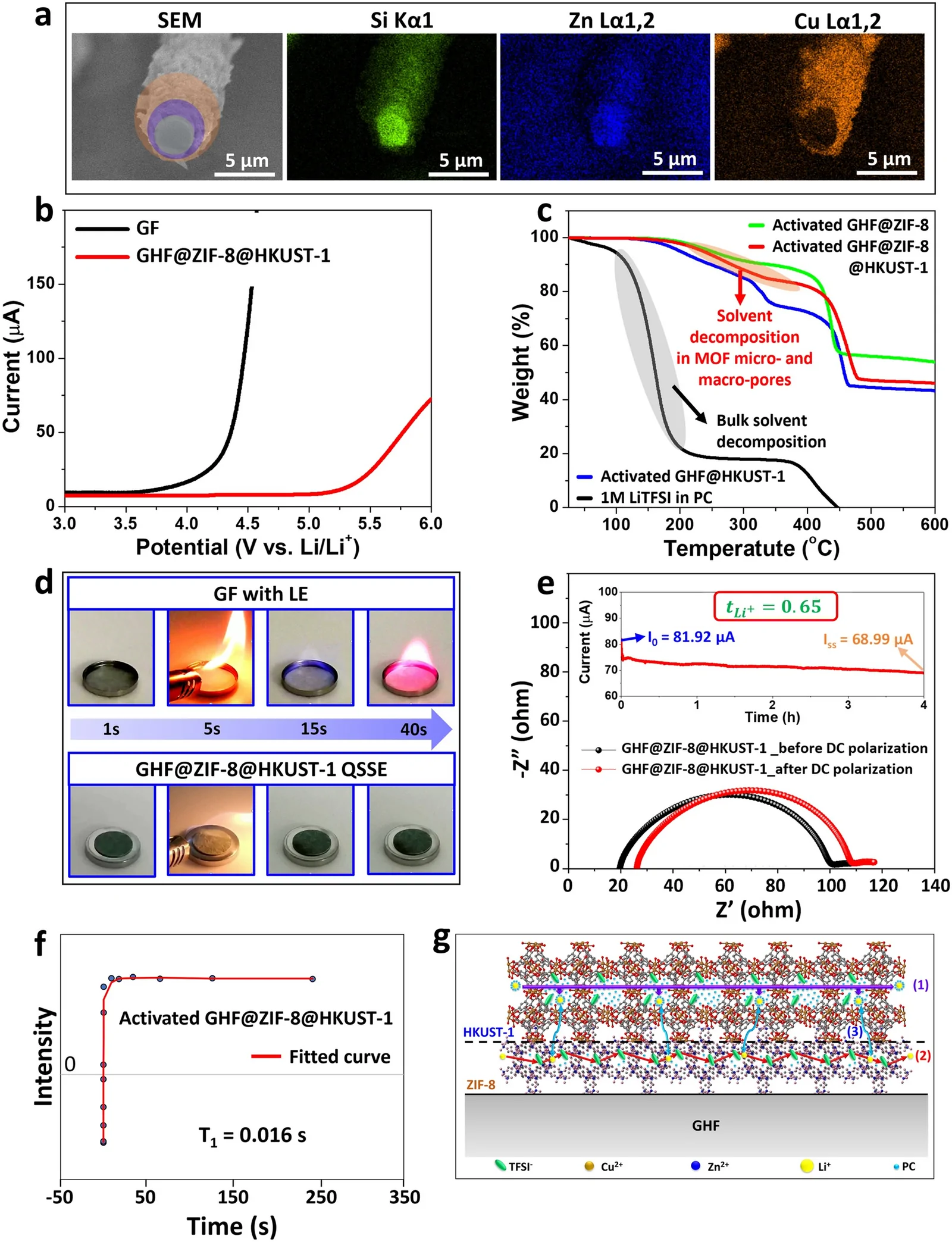
Ion transport, electrochemical, and thermal properties of core–shell MOF grown on GHF. **a** Cross-sectional SEM image of ZIF-8@HKUST-1 core–shell grown on single glass-based hybrid fiber and its corresponding EDS elemental mapping images. **b** Linear sweep voltammetry (LSV) curves of GF and GHF@ ZIF-8@HKUST-1 QSSE. **c** Thermogravimetric analysis (TGA) curves of the typical liquid electrolyte (1 M LiTFSI in PC) and different activated GHF@MOF separators. **d** Combustion tests of GF in LE (1 M LiTFSI in PC) and GHF@ ZIF-8@HKUST-1 quasi-solid electrolyte. **e** Nyquist plots of the Li//Li symmetric cell of GHF@ ZIF-8@HKUST-1 QSSE before and after DC polarization (the insets are the current–time curves at 10 mV polarization). **f** Inversion recovery plots, fitted curves, and calculated T1 of GHF@ ZIF-8@HKUST-1 QSSE. **g** Faster and more efficient ionic transport through multiple pathways in core–shell MOF grown on GHF. ((1), (2), and (3) are three possible Li ionic pathways in GHF@ ZIF-8@HKUST-1 QSSE)

To leverage the benefits and address the limitations of both MOF-based QSSEs (see Note S9 for the detailed discussion), we developed a novel core–shell MOF structure grown on GHF, with ZIF-8 as the core and HKUST-1 as the shell (Figs. S8–S10; see Note S10 for the detailed discussion). The QSSE based on GHF@ZIF-8@HKUST-1 exhibited the clearly defined core–shell configuration and the large electrochemical window (Fig. 5a, b). Moreover, it demonstrated excellent thermal stability (Figs. S11 and S12; see Note S11 for the detailed discussion), with the solvent decomposition temperature within the pores ranging from above 200 °C to nearly 400 °C, which is better than the GHF@HKUST-1 structure and only slightly lower than the GHF@ZIF-8 QSSE (Fig. 5c). The advantage of this core–shell structure lies in its ability to store both the solvated and de-solvated configurations of Li^+^ after activation, as evidenced by the clear presence of Li^+^-PC-TFSI^−^ and Li^+^-TFSI^−^-MOF interactions, represented by two new peaks observed in solid NMR, Raman, and FTIR spectra (Fig. S13). This not only helps reduce resistance at the electrolyte/electrode interface through the wettability of the electrode surface by the HKUST-1 shell containing solvated Li^+^, but also ensures low activation energy (0.123 eV) and high ionic conductivity at room temperature (2.43 × 10^–4^ S cm^−1^) (Fig. S14) and high thermal stability due to the ZIF-8 core, which has a high density of de-solvated Li^+^ and strong binding energy between PC and Zn^2+^. As a result, it displayed superior flame resistance compared to the GF in conventional liquid electrolytes (Fig. 5d and Videos S1 and S2). This enhanced flame resistance is attributed to the stability of LE components being confined within the MOF pores. The exceptional thermal stability of our quasi-solid-state electrolyte effectively prevents combustion and short circuiting in batteries, especially when operating at high temperatures. Another notable feature of the core–shell QSSE is the enhancement of the Li^+^ transference number, reaching up to 0.65 (Fig. 5e), and the increased mobility of Li^+^, as evidenced by the T1 value in solid NMR measurements dropping to just 0.016 s (Fig. 5f). This can be explained by the presence of multiple channels for Li^+^ ion transport within the core–shell MOF structure (Fig. 5g). In addition to the two main continuous ion conduction channels (1) and (2), corresponding to solvated Li^+^ diffusion in the large channel of HKUST-1 and de-solvated Li^+^ hopping, ion transport can also occur at the interface between these two structures (Figs. S15, S16, and Table 2; see Note S12 for the detailed discussion).

### Application of Prepared QSSEs in LMBs at Extremely High Temperature

**Fig. 6:**
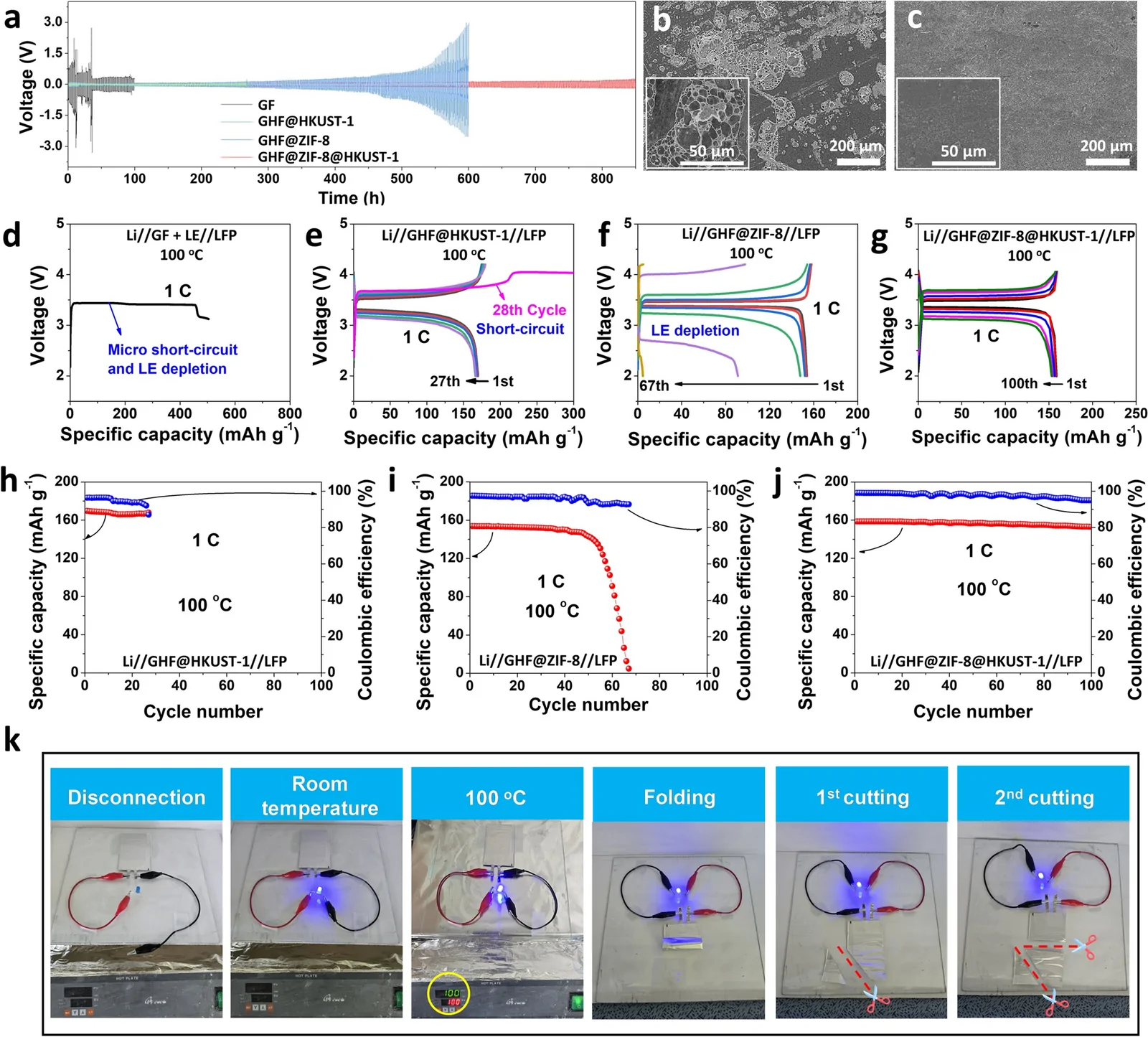
Li//Li symmetric cells and Li//LFP cells utilizing MOF-based QSSEs. **a** Galvanostatic cycling of Li//Li symmetric cells assembled with conventional GF membrane combined with typical liquid electrolyte (LE) and different GHF@MOF quasi-solid electrolyte at current densities of 1 mA cm^−2^ at room temperature (RT). SEM images of Li metal surfaces detached from the Li//Li symmetric cell using **b** commercial GF in LE and **c** GHF@ ZIF-8@HKUST-1 QSSE after 100 h. The cyclic performance of Li//LFP cells at extremely high temperature (100 °C) and 1 C rate using **d** GF in LE and QSSEs prepared from (**e, h**) GHF@HKUST-1, (**f, i**) GHF@ZIF-8, and (**g, j**) GHF@ZIF-8@HKUST-1. **k** Illustration of graphite//GHF@ ZIF-8@HKUST-1 QSSE//LFP pouch cells for lighting LED lamp in various harsh conditions

The Li plating/stripping behavior through these QSSEs was investigated in Li//Li symmetric cells (Fig. 6a). Notably, the core–shell QSSE sustained long-term cycling stability for over 850 h without short circuiting or significant voltage polarization (Fig. S17; see Note S13 for the detailed discussion). The Li metal surface remained smooth after 100 h in the core–shell QSSE-based cell, while high-density, disordered Li dendrites were observed in cells with commercial GF separators and LE (Fig. 6b, c). It is attributed to its uniform and stable ion flow through nano-sized Li^+^ transport channels in the ordered porous structure on GHF, effectively preventing Li dendrite formation. Thus, the core–shell QSSE demonstrated outstanding electrochemical stability, enhanced Li^+^ ion transport efficiency, and superior dendrite-blocking capability. Furthermore, Li//LFP cells tested at room temperature showed stable cycling for 100 cycles with Coulombic efficiencies above 99%, confirming the reliability of MOF-based QSSEs under mild operating conditions (Figs. S18–S20; see Note S14 for the detailed discussion). The Li//GHF@ZIF-8@HKUST-1 QSSE//LFP cell delivered a discharge capacity of around 149 mAh g^−1^ after 100 cycles, which was 1.16 times higher than that of the GF + LE cell (approximately 128 mAh g⁻^1^) (Fig. S18). The voltage polarization of the Li//GF + LE//LFP cell exceeded 170 mV after 10 cycles, nearly 1.4 times higher than that of the QSSE (~ 120 mV) (Fig. S19), indicating severe interfacial polarization and unstable SEI evolution. Furthermore, EIS analyses presented revealed that the charge-transfer resistance of the GF + LE cell (~ 42 Ω) was 1.6 times higher than that of the GHF@ZIF-8@HKUST-1 QSSE, which was around 26 Ω (Fig. S20). This confirms the enhanced interfacial kinetics and lower energy barrier for Li^+^ transfer induced by MOF confinement.

Importantly, this QSSE also exhibited excellent thermal stability and flame resistance, ensuring safe and stable operation even at high operating temperatures. To confirm this, we assembled Li//LFP batteries with the prepared QSSEs and operated them at an extremely high temperature (100 °C) at a 1 C rate. Batteries using conventional liquid electrolyte (1 M LiTFSI in PC) and commercial GF separators were also tested for comparison. As shown in Fig. 6d, the GF + LE cell failed immediately at 100 °C, delivering only about 100 mAh g⁻1 before short circuiting. This failure is attributed to the significant decomposition of the bulk LE (1 M LiTFSI in PC) at temperatures exceeding 100 °C (TGA results, Fig. 2e). Similar to the Li//Li symmetric cells, the Li//LFP cells using single MOF-based QSSEs performed better due to the confinement of the liquid electrolyte within the nano-sized MOF pores. However, these cells still exhibited certain weaknesses. The GHF@HKUST-1, with its large pore size, accommodated most of the solvated Li^+^ ions within these pores. These solvated Li^+^ ions migrate to the electrode and undergo a de-solvation process at the electrolyte/electrode interface. This process is slow due to strong interactions between the PC solvent and Li^+^ (as demonstrated in DFT simulations), causing solvated Li^+^ to accumulate near the electrode surface, leading to non-uniform deposition and dendrite formation. As a result, the Li//LFP cell with GHF@HKUST-1 QSSE short circuited after only 28 cycles (Fig. 6e, h). In contrast, the majority of de-solvated Li^+^ structures found in the small pores of ZIF-8 do not contain solvation shells, enabling more direct and uniform interaction with the electrode surface. This promotes layer-by-layer Li plating rather than random dendritic growth. Additionally, the de-solvated Li^+^ ions interact with the solid-state electrolytes to form robust SEI layers, which act as an effective dendrite-blocking barrier. However, the ability to retain low amounts of PC solvent in the small ZIF-8 pores, combined with the strong binding energy between the PC solvent and Zn^2+^ (as confirmed by DFT simulations), limits electrode/electrolyte interfacial wetting (Fig. S21; see Note S15 for the detailed discussion), leading to increased cell resistance and rapid capacity loss. Consequently, the cell assembled with GHF@ZIF-8 showed increased voltage polarization and rapid capacity degradation after only 50 cycles (Fig. 6f, i). Notably, the combination of solvated and de-solvated Li^+^ structures in the GHF@ZIF-8@HKUST-1 QSSE delivered exceptional performance, ensuring stable operation of the Li//LFP battery even at extremely high operating temperatures (100 °C) with the capacity retention reaching 97% of the initial discharge specific capacity value (158.7 mAh g⁻^1^) after 100 cycles at 1 C rate (Fig. 6g, j). Issues related to short circuiting and interfacial wetting were effectively addressed through the synergistic effects of this core–shell structure (Figs. S21 and S22; see Notes S15 and S16 for the detailed discussion) and the uniform Li^+^ transport through multiple continuous ionic conduction channels (see Note S12). Post-cycling SEM analyses further supported these results: Dense dendrites were observed on Li cycled with GHF@HKUST-1, smoother but depleted surfaces appeared with GHF@ZIF-8, while GHF@ZIF-8@HKUST-1 preserved the most uniform Li morphology (Fig. S23; see Note S17 for the detailed discussion). In addition, stability analysis confirmed that all GHF@MOF membranes maintained their structural integrity after prolonged cycling at 100 °C, indicating that the superior performance originates from interfacial processes rather than membrane degradation (Fig. S24; see Note S17 for the detailed discussion). Furthermore, rate performance tests at 100 °C confirmed that the core–shell QSSE maintained high discharge capacities across a wide range of current densities, with nearly full recovery when the rate was returned to 0.1 C (Fig. S25; see Note S18 for the detailed discussion). This demonstrates the robustness and reversibility of the MOF-based electrolyte under high-rate, high-temperature conditions.

To demonstrate the scalability of our QSSE design, a pouch cell was fabricated, delivering a total output capacity of 6.80 mAh (Fig. S26; see Note S19 for the detailed discussion), nearly an order of magnitude higher than that of a coin cell counterpart (0.78 mAh). Additionally, we captured and displayed digital images of the pouch cell with an LED light in Fig. 6k. The LED continued to shine brightly without any signs of short circuiting, even under extreme conditions such as heating at 100 °C, bending, and cutting. This demonstrates the high reliability and safety of the pouch cell based on the prepared QSSE, highlighting its significant potential for practical applications in quasi-solid-state LMBs in future research.

## Conclusions

In summary, we present a direct scientific evidence for confining Li solvation in nanopores through 1-D MOF-based model study with comprehensive experimental analysis and neural network potential-based simulation modeling. Accordingly, we suggest an innovative MOF-based QSSE featuring a hierarchical core–shell structure, where a ZIF-8 core and HKUST-1 shell are engineered onto a GHF separator, forming multiple continuous ion-conducting pathways that significantly enhance Li⁺ transport efficiency, thermal resilience, and electrochemical stability. The ZIF-8 core facilitates rapid Li⁺ de-solvation, while the HKUST-1 shell stabilizes solvated Li⁺, enabling efficient ion conduction, uniform Li⁺ migration, and dendrite suppression. As a result, the QSSE exhibits a high ionic conductivity of 2.43 × 10⁻^4^ S cm⁻^1^, a lithium transference number of 0.65, thermal stability exceeding 200 °C, and a wide electrochemical window of ~ 5.3 V, ensuring excellent safety and long-term durability. When paired with a LiFePO₄ (LFP) cathode, the QSSE exhibits 97% capacity retention after 100 cycles at 100 °C, demonstrating exceptional performance under extreme conditions. This study provides fundamental insights into lithium-ion solvation and transport within MOF architectures, paving the way for scalable, high-performance QSSE designs.

## Supplementary Information

Below is the link to the electronic supplementary material.

Supplementary file1 (DOCX 28816 KB)

Supplementary file2 (MP4 5834 KB)

Supplementary file3 (MP4 3528 KB)
