# Correction: Confining Li^+^ Solvation in Core–Shell Metal–Organic Frameworks for Stable Lithium Metal Batteries at 100 °C

**DOI:** 10.1007/s40820-026-02096-w

**Published:** 2026-03-11

**Authors:** Minh Hai Nguyen, Jeongmin Shin, Mee-Ree Kim, Quan Van Nguyen, JinHyeok Cha, Sangbaek Park

**Affiliations:** 1 Department of Materials Science and Engineering, Chungnam National University, Daejeon, 34134 Republic of Korea; 2 School of Mechanical Engineering, Chonnam National University, Gwangju, 61186 Republic of Korea

**Correction to: Nano-Micro Lett. (2026) 18:135** 10.1007/s40820-025-01988-7

Following publication of the original article [1], the authors noticed that Fig. 2 was published with an incorrect panel order, which does not reflect the final intended version approved during the proof stage. As a result, the panel sequence in Fig. 2 is inconsistent with the figure caption and manuscript text. This issue is limited strictly to the order and labeling of the figure panels. The experimental data, scientific interpretation, results, and conclusions of the paper remain completely unchanged.

The correct Fig. 2 has been provided in this Correction.

The incorrect Fig. 2 is:

**Fig. a:**
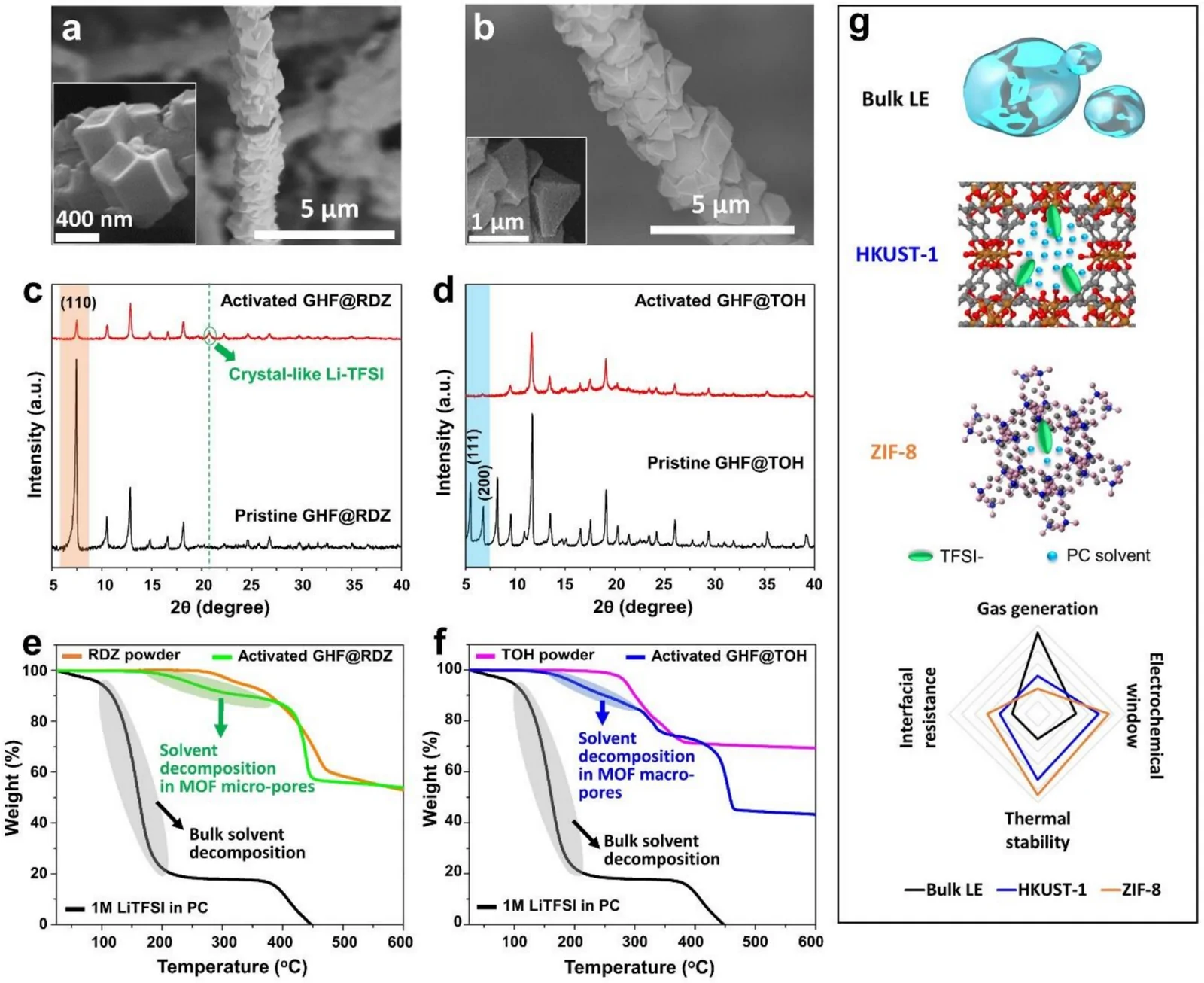
Morphologies of single MOFs grown on GHF and liquid electrolyte infiltration. SEM images of as-synthesized **a** truncated octahedral HKUST-1 and **b** rhombic dodecahedral ZIF-8 particles grown on GHF. XRD patterns of pristine and activated **c** GHF@HKUST-1 and **d** GHF@ ZIF-8. Thermogravimetric analysis (TGA) curves of activated **e** GHF@HKUST-1 and **f** GHF@ZIF-8 separators in comparison with the typical liquid electrolyte and corresponding MOF powders. While the conventional LE (1 M LiTFSI in PC) showed poor thermal stability (highlighted in gray), the activated MOF-based QSSEs exhibited significantly enhanced thermal stability. The activated GHF@ZIF-8 revealed a slower decomposition rate over a broader temperature range (marked in green) compared to activated GHF@HKUST-1 (marked in blue). **g** Schematic depiction of the limitations of conventional liquid electrolytes and the features of MOF-based semi-solid electrolytes with varying pore sizes

The correct Fig. 2 is:

**Fig. 2:**
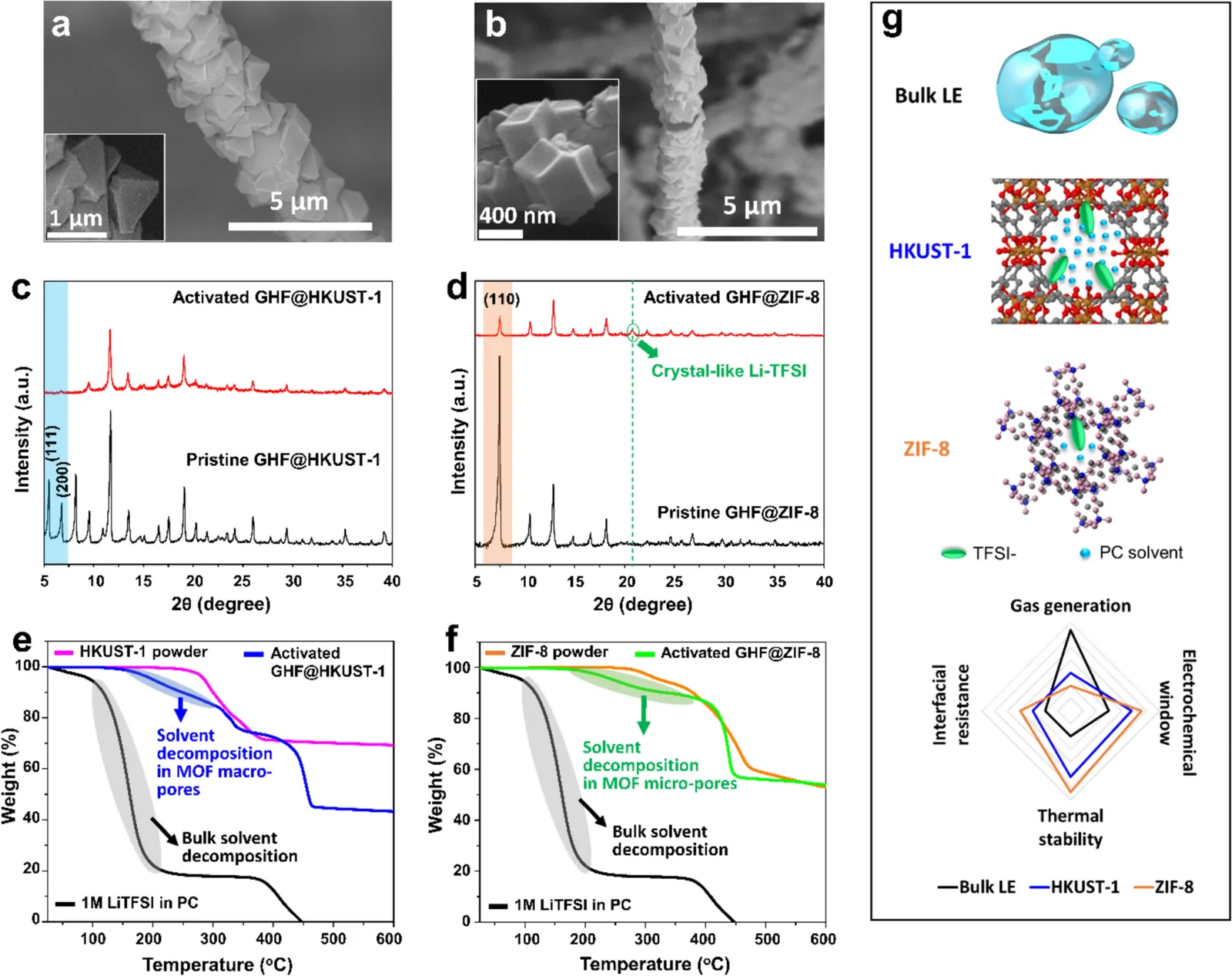
Morphologies of single MOFs grown on GHF and liquid electrolyte infiltration. SEM images of as-synthesized **a** truncated octahedral HKUST-1 and **b** rhombic dodecahedral ZIF-8 particles grown on GHF. XRD patterns of pristine and activated **c** GHF@HKUST-1 and **d** GHF@ ZIF-8. Thermogravimetric analysis (TGA) curves of activated **e** GHF@HKUST-1 and **f** GHF@ZIF-8 separators in comparison with the typical liquid electrolyte and corresponding MOF powders. While the conventional LE (1 M LiTFSI in PC) showed poor thermal stability (highlighted in gray), the activated MOF-based QSSEs exhibited significantly enhanced thermal stability. The activated GHF@ZIF-8 revealed a slower decomposition rate over a broader temperature range (marked in green) compared to activated GHF@HKUST-1 (marked in blue). **g** Schematic depiction of the limitations of conventional liquid electrolytes and the features of MOF-based semi-solid electrolytes with varying pore sizes

The original article [1] has been corrected.
