# Supplementary material for: Confining Li⁺ Solvation in Core–Shell Metal–Organic Frameworks for Stable Lithium Metal Batteries at 100 °C
Source: Nanomicro Lett. 2026 Jan 5;18:135. doi: 10.1007/s40820-025-01988-7 (PMC12765793; doi:10.1007/s40820-025-01988-7)
Supplement: Supplementary file 1 — Supplementary file1 (DOCX 28816 KB) [file 40820_2025_1988_MOESM1_ESM.docx]

Supporting Information for

**Confining Li⁺ Solvation in Core-Shell Metal-Organic Frameworks for Stable Lithium Metal Batteries at 100 °C**

Minh Hai Nguyen^1^, Jeongmin Shin^2^, Mee-Ree Kim^1^, Quan Van Nguyen^1^, JinHyeok Cha^2,^*, and Sangbaek Park^1,^*

^1^Department of Materials Science and Engineering, Chungnam National University, Daejeon 34134, Republic of Korea

^2^School of Mechanical Engineering, Chonnam National University, Gwangju 61186, Republic of Korea

* Corresponding authors. E-mail: [sb.park@cnu.ac.kr](mailto:sb.park@cnu.ac.kr) (Sangbaek Park); [jinhyeok.cha@chonnam.ac.kr](mailto:jinhyeok.cha@chonnam.ac.kr) (JinHyeok Cha)

Supplementary Figures and Tables


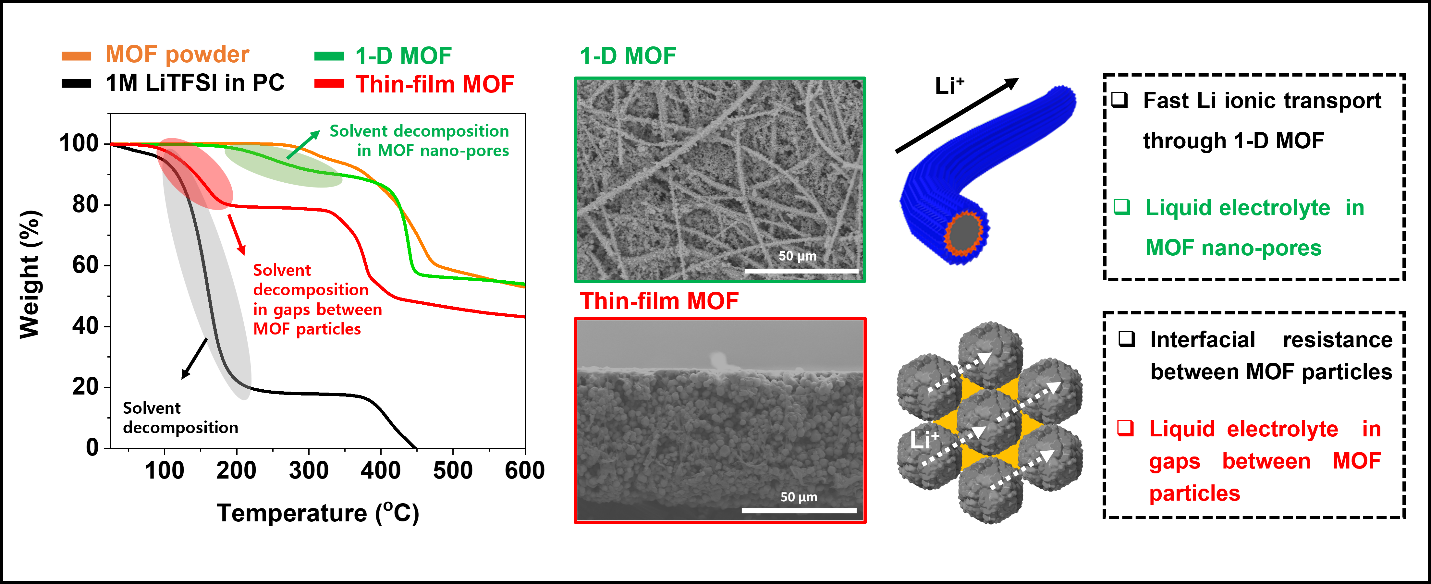


**Fig. S1** Comparison between MOF thin-film-based QSSE (previous study) [S1] and 1D MOF-based QSSE (this work)

**Note S1 Fabrication of various MOFs grown on GHF**

To eliminate structural and morphological variations that could influence our results, different morphologies of ZIF-8 and HKUST-1 were synthesized on a glass-based hybrid fiber (GHF) substrate. The surface of commercial glass fiber (GF) is relatively inert [S2–S4], making it unsuitable for the nucleation and growth of metal-organic frameworks (MOFs). As observed in a control synthesis of rhombic dodecahedral ZIF-8 (RDZ) and on commercial GF, under the same conditions as those used for modified fibers, the surface remained largely smooth, with only a few sparsely distributed MOF crystallites (Figs. S2a-c). This limited growth can be attributed to the lack of functional groups on the inert GF surface. To overcome this, we engineered a glass-based hybrid fiber (GHF) by integrating bovine serum albumin (BSA) with commercial GF, using (3-aminopropyl) triethoxysilane (APTES) and glutaraldehyde (GA) as coupling agents. Biological fibers are well known for being rich in functional groups such as amines and carboxylates [S5–S7], which can facilitate the nucleation and growth of nanomaterials. Among these, BSA is a particularly promising candidate due to its abundance of –NH₂, –COOH, and –OH groups [S8], which strongly coordinate with metal ions and promote uniform MOF crystallization. Unlike other biological materials such as silk, gelatin, or collagen, BSA offers high water solubility and low cost [S9–S11], making it an ideal modifier for scalable and controllable surface functionalization of GF to improve MOF adhesion and structural stability. The resulting GHF surfaces, enriched with functional groups, significantly enhanced MOF growth with nearly complete surface coverage (Figs. S2d-i). By tuning the precursor concentrations, we were able to selectively control the morphology of both ZIF-8 and HKUST-1 on GHF. Specifically, rhombic dodecahedral ZIF-8 (RDZ), sheet-flower-like ZIF-8 (SFZ), and leaf-like ZIF-8 (LZ) were obtained at salt-to-linker molar ratios of 1/77, 1/15, and 1/10, respectively. Similarly, nanoflower HKUST-1 (NFH), truncated octahedral HKUST-1 (TOH), and cubic HKUST-1 (CH) were synthesized at salt/linker/additive molar ratios of 1.8/1/50, 1.8/1/100, and 1.8/1/150, respectively.

**Fig. S2 a,** Schematic illustration of MOFs on the inert surface of commercial glass fiber (GF). **b,c,** SEM image of bare GF membrane after MOF growth process. **d-f**, Schematic illustrations and SEM images of as-synthesized ZIF-8 particles grown on the glass-based hybrid fiber (GHF) with various morphologies: Rhombic dodecahedral ZIF-8 (RDZ) (**d**), Sheet-flower-like ZIF-8 (SFZ) (**e**) and Leaf-like ZIF-8 (LZ) (**f**). **g-i**, Schematic illustrations and SEM images of as-synthesized HKUST-1 particles grown on GHF with various morphologies: Nanoflower HKUST-1 (NFH) (**g**), Truncated octahedral HKUST-1 (TOH) (**h**), and Cube HKUST-1 (CH) (**i**)

**Note S2 Structural and electrochemical analysis of various MOFs on GHF**

Structural and electrochemical stability analyses confirmed that RDZ and TOH were the most suitable representatives of ZIF-8 and HKUST-1, respectively, for our MOF-based QSSEs (Fig. S3). X-ray diffraction (XRD) analysis of various ZIF-8-based MOFs grown on GHF confirmed their highly crystalline nature. Among them, the GHF@RDZ sample exhibited good agreement with the standard diffraction peaks of ZIF-8, while other ZIF-8-derived structures showed notable deviations (Fig. S3a). In particular, GHF@RDZ demonstrated a significantly more intense peak at a low 2θ angle compared to the other morphologies, indicating enhanced structural order. In contrast to the ZIF-8-based samples, all HKUST-1-derived MOFs displayed relatively consistent diffraction patterns that matched well with standard HKUST-1 peaks (Fig. S3b). Nonetheless, the (111) reflection at the lowest 2θ value was most intense for the GHF@TOH sample, suggesting a high degree of orientation or pore alignment. The prominent low-angle XRD peaks in both GHF@RDZ and GHF@TOH suggest a higher pore density and better pore accessibility compared to the other structures, making them strong candidates for quasi-solid-state electrolytes (QSSEs) with enhanced liquid electrolyte (LE) uptake.

Electrochemical stability evaluations further revealed that QSSEs based on GHF@MOFs exhibited wider electrochemical windows than conventional glass fiber (GF) with standard LE (1M LiTFSI in PC), particularly for GHF@RDZ and GHF@TOH systems (Fig. S3c-d). This improvement can be attributed to the strong anion-trapping ability of RDZ and TOH morphologies, especially for bulky anions like TFSI⁻, which possess high HOMO energy levels (approximately –6.0 to –7 eV vs. vacuum) [S12, S13] and are prone to oxidation at elevated voltages. Moreover, these MOFs feature spatial confinement, abundant open metal sites, and polar functional groups that can engage in electrostatic or coordination interactions with the anions and solvents of the confined LE. Such interactions draw electron density away from the molecular orbitals, lowering the HOMO level and thereby reducing the susceptibility of the system to oxidative degradation. This leads to improved oxidative stability and an expanded operational voltage window of the resulting QSSEs. In contrast, conventional LE systems, where TFSI⁻ and solvent molecules (e.g., PC) remain uncoordinated, retain high HOMO levels and are more vulnerable to oxidation at high voltages. Based on these findings, we selected RDZ and TOH as representative MOF structures for ZIF-8 and HKUST-1, respectively, for further in-depth investigation of lithium-ion solvation structures, Li⁺ transport kinetics, and the development of core-shell MOF structures on GHF.

**Fig. S3 a, b,** XRD patterns of different GHF@ZIF-8 (**a**) and GHF@HKUST-1 (**b**) membranes. **c,d,** Linear sweep voltammetry (LSV) curves of various GHF@ZIF-8 (**c**) and GHF@HKUST-1 (**d**) QSSEs compared to GF in typical LE (1M LiTFSI in PC)

**Note S3 Infiltrated amounts of liquid electrolyte in GHF@MOFs**

To quantify the amount of liquid electrolyte (LE) confined within the MOF pores, we carefully analyzed the mass evolution of GHF@MOF membranes during the activation process. The pristine GHF mass was first measured, and the MOF loading was obtained from the weight increase after MOF growth. Following thermal and electrochemical activation, the membranes were thoroughly washed and dried at 60 °C for 24 h to remove any residual or externally adsorbed electrolyte. The content of Li salt inside the pores was then estimated from the weight loss above 400 °C in the TGA curves, which corresponds to LiTFSI decomposition. By subtracting the contributions of both MOF loading and Li salt from the final membrane mass, the actual amount of confined electrolyte was accurately determined. This method excludes errors arising from surface-adsorbed solvent, thereby providing a more reliable evaluation of the true electrolyte uptake in the MOF nanochannels.

As summarized in Supplementary Table 1, GHF@HKUST-1 exhibited a higher confined LE content (1.02 mg cm⁻²) than GHF@ZIF-8 (0.96 mg cm⁻²), consistent with the larger apertures of HKUST-1 (~0.9 nm) that can accommodate more solvated species. In contrast, the narrow pores of ZIF-8 (~0.54 nm) restricted solvent infiltration, leading to a lower confined electrolyte content. The core–shell GHF@ZIF-8@HKUST-1 achieved an intermediate value of 1.62 mg cm⁻² with a confined LE/MOF ratio of 0.1731, representing a synergistic balance: the HKUST-1 shell supplied sufficient solvent for interfacial wetting, while the ZIF-8 core ensured strong confinement and promoted desolvated Li⁺ transport. Notably, these electrolyte amounts are much smaller than that of conventional GF separators soaked in liquid electrolyte (~60 mg cm⁻²), underscoring the effectiveness of MOF nanochannels in achieving low-electrolyte-content QSSEs with enhanced thermal stability and high energy density.

**Table S1** Loading mass of liquid electrolyte (LE) in pores of different MOF-based QSSEs

| **Sample** | **Mass of pristine samples (mg cm^-2^)** | **Mass of loaded MOFs (mg cm^-2^)** | **Mass of MOF-based QSSEs (mg cm^-2^)** | **Weight percentage of Li salt remained in QSSEs (%)** | **Mass of LE in pores (mg cm^-2^)** | **Confined LE/MOF ratios** |
| --- | --- | --- | --- | --- | --- | --- |
|  | **(A)** | **(B = A – A1)** | **(C)** | **(D)** | **(E = C - A - C*D/100)** | **(F = E/B)** |
| GHF | 3.98 | - | - | - | - | - |
| GHF@ZIF-8 | 9.77 | 5.79 | 15.49 | 30.70 | 0.96 | 0.1666 |
| GHF@HKUST-1 | 9.52 | 5.54 | 14.74 | 28.50 | 1.02 | 0.1840 |
| GHF@ZIF-8@HKUST-1 | 13.35 | 9.37 | 22.38 | 33.10 | 1.62 | 0.1731 |

**Note S4 The characteristics of QSSEs based on single MOF-coated GHF**

The characteristics of our MOF-based QSSEs in comparison with conventional LE are illustrated in Fig. 2g. Traditional LEs exhibit low electrode/electrolyte interfacial resistance due to their high ionic conductivity; however, they suffer from low boiling points, a limited electrochemical window, and the necessity for large electrolyte volumes in batteries. These factors make them prone to decomposition, particularly at elevated temperatures and high voltages, leading to solvent evaporation and gas evolution inside the battery, which compromises safety and increases the risk of explosion. In contrast, when confined within MOF pores, LE exhibits significantly improved thermal and electrochemical stability compared to its bulk state. RDZ, with its small pore size of 0.54 nm (Fig. S4a), effectively retains a minimal amount of LE within individual pores, resulting in a high decomposition energy requirement. Consequently, RDZ demonstrates excellent thermal and electrochemical stability while minimizing gas evolution during operation, even at high working temperatures. However, due to its highly closed structure, solvent leakage onto electrode surfaces is limited, leading to high interfacial resistance and low ionic conductivity. Conversely, TOH, with its larger pore size of 0.9 nm (Fig. S4b), facilitates solvent migration onto electrode surfaces, thereby reducing electrode/electrolyte interfacial resistance and enhancing ionic conductivity. However, its thermal and electrochemical stability is lower than that of RDZ due to the weaker confinement effect.


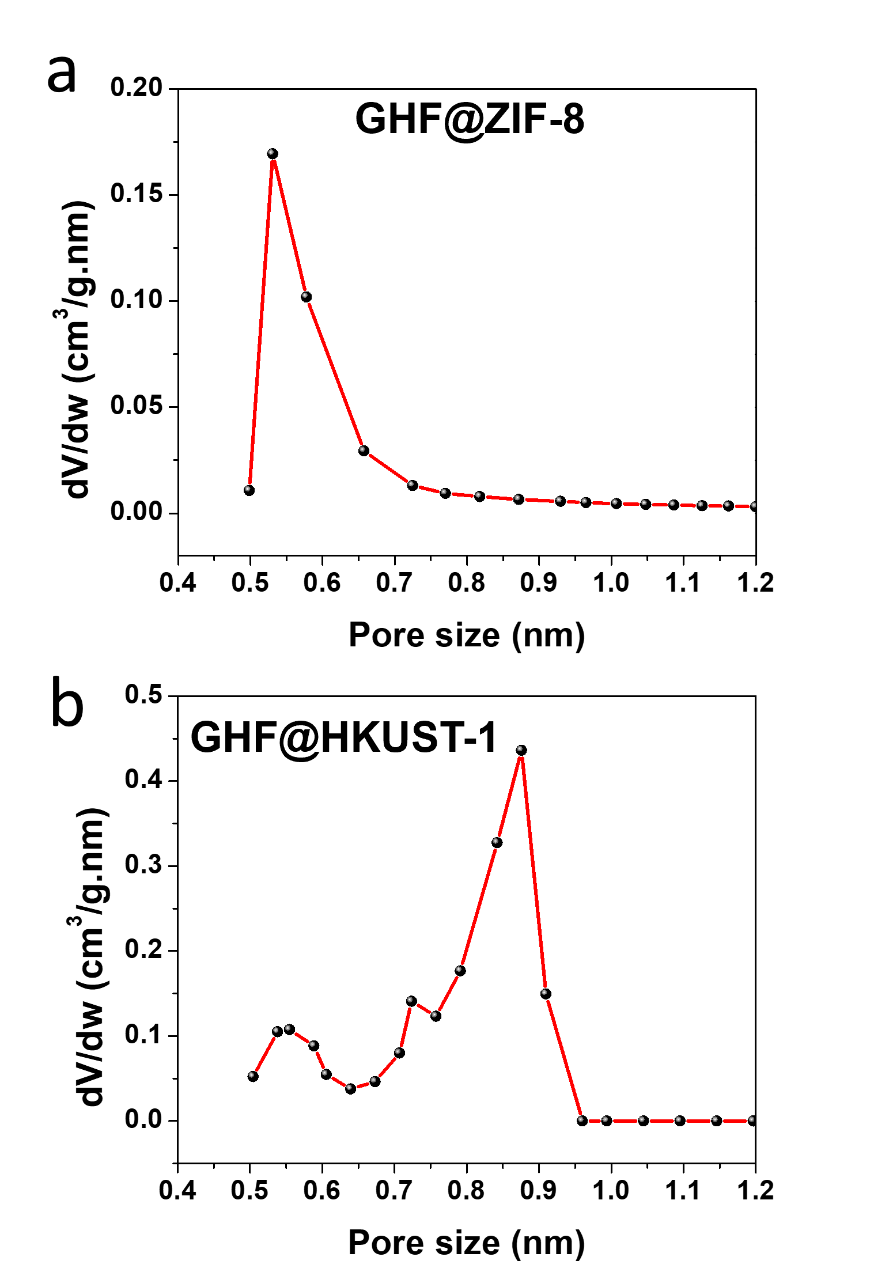


**Fig. S4 a, b,** Pore size distributions of GHF@ZIF-8 (**a**) and GHF@HKUST-1 (**b)**

**Note S5 Raman analysis for the investigation of solvation structures and ion-transport properties in GHF@MOF QSSEs**

Raman spectroscopy was employed as a preliminary tool to explore the chemical interactions of LiTFSI liquid electrolyte (LE) within MOF pores, as well as the ion transport dynamics of Li^+^ within the MOF nanochannels (Fig. 3a). For bulk LE (1M LiTFSI in PC), a strong characteristic peak at 707 cm^-1^ was observed, representing Li^+^-PC interactions in LE [S16], while another peak at 474 cm^-1^ corresponded to C=O bending and ring deformation of PC molecules [S17]. Upon activation to confine LE within HKUST-1 pores, the presence of a strong peak at 976 cm^-1^ and a weak peak at 223 cm^-1^ indicated that Li^+^-PC-TFSI^-^ interactions dominated over Li^+^-TFSI^-^-MOF interactions in the large pores of HKUST-1. This suggests that PC within HKUST-1 pores exhibits enhanced affinity toward Li^+^, forming a solvation structure in which Li^+^ is surrounded by multiple PC molecules, thereby weakening the Li^+^-TFSI^-^ bond. Interestingly, ZIF-8, with its smaller pores, exhibited significantly enhanced Li^+^-TFSI^-^-MOF interactions (peak at 372 cm^-1^), while the Li^+^-PC-TFSI^-^ interactions were substantially reduced. This is attributed to strong nanoscale confinement effects, wherein Li^+^ and TFSI^-^ are brought closer together in the narrow space, leading to the formation of a crystal-like Li-TFSI structure, as evidenced by the emergence of a new XRD peak (Fig. 2c). These findings indicate that a de-solvated Li^+^ structure dominates within the narrow pore space of ZIF-8. Raman shifts at 748 cm^-1^ correspond to Li^+^-TFSI^-^ interactions in unactivated LiTFSI salt crystals, which still exist within the GHF@MOF membrane body after post-drying. These residual salts serve as a reference, emphasizing the distinct Li⁺–TFSI⁻ interactions occurring both inside and outside the MOF pores.

**Note S6 The difference in ionic activation energy of GHF@MOF-based QSSEs**

To further analyze and compare ion transport behavior within different single-MOF nanochannels, we conducted electrochemical impedance spectroscopy (EIS) measurements of QSSEs based on GHF@ZIF-8 and GHF@HKUST-1 over a temperature range from room temperature (25 °C) to 70 °C. The ionic activation energies were then calculated using Arrhenius plots, as shown in Fig. S5. The linearity of all Arrhenius plots indicates that no structural or compositional changes occurred in the MOF-based separators during measurement, confirming the thermal stability of these QSSEs within the 25–70 °C range. Notably, the activation energy of the GHF@ZIF-8-based QSSE (0.106 eV) was significantly lower than that of the GHF@HKUST-1-based QSSE (0.228 eV), suggesting a more favorable Li⁺ transport mechanism in the former. This difference implies that the interfacial de-solvation process of Li⁺ ions is energetically more efficient in the ZIF-8-based system. The confined and rigid nanochannels of ZIF-8 facilitate the preferential transport of partially or fully de-solvated Li⁺ ions toward the electrode interface. Consequently, these de-solvated Li⁺ ions can immediately participate in electron transfer to form metallic lithium without requiring an additional de-solvation step. In contrast, the larger and less confined HKUST-1 channels allow Li⁺ ions to remain heavily solvated by PC molecules during transit. Upon reaching the electrode surface, these solvated Li⁺ ions must undergo a further de-solvation step to remove the surrounding solvent shell before electron transfer can proceed, resulting in a higher energy barrier and slower interfacial kinetics.

**
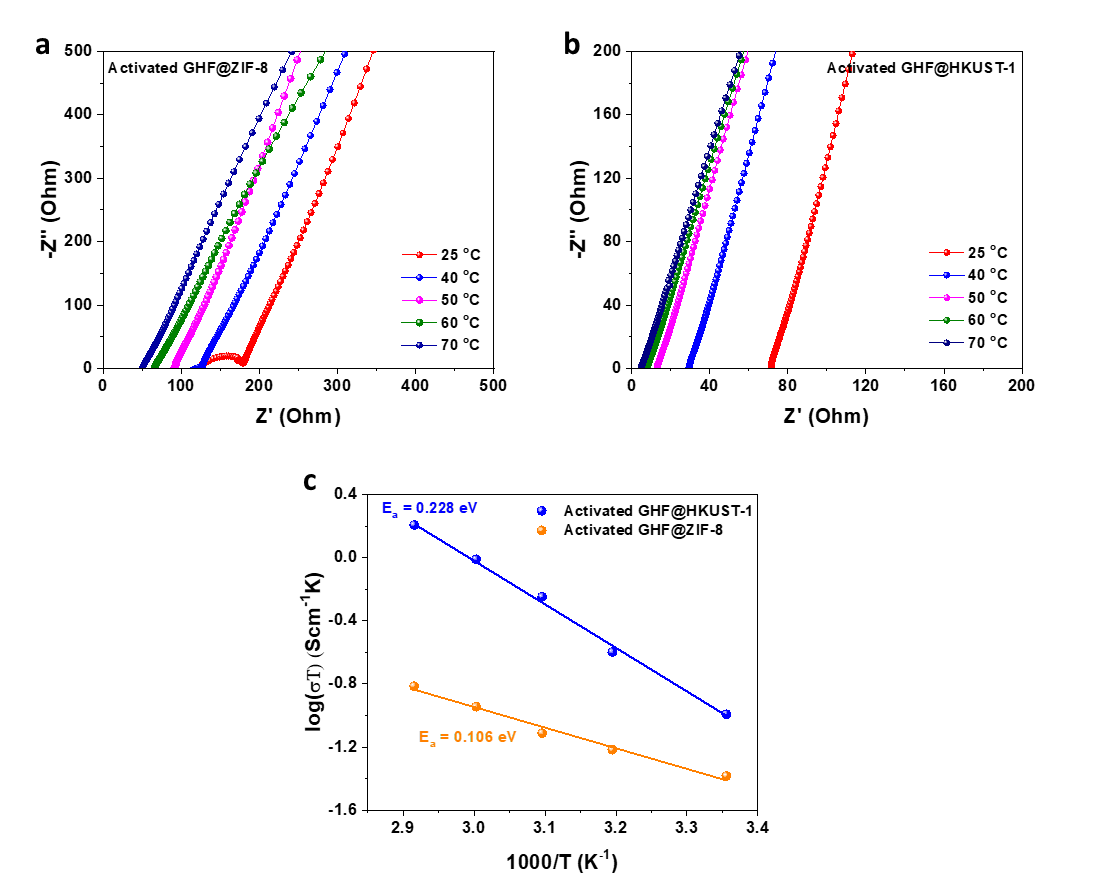
**

**Fig. S5 a-c,** Nyquist plots of activated GHF@ZIF-8 (**a**) and GHF@HKUST-1 (**b**) at various temperatures from 25 to 70 ^o^C, and their corresponding Arrhenius plots (**c**)

**Note S7 DFT binding energies of MOF-TFSI^-^ and MOF-PC**

To elucidate the solvation structures of Li⁺ ions within different MOF nanochannels, we calculated the binding energies of relevant species both inside and outside the MOF pores using density functional theory (DFT). To simplify the simulations, the pore structures of TOH and RDZ were modeled based on the representative pore clusters of HKUST-1 and ZIF-8, respectively (Figs. S6a, b). In this analysis, Li⁺ ions (purple spheres) were placed either in the external environment (Figs. 4a,d) or confined within MOF pores (Figs. 4b, c, e, f), in the presence of either only TFSI⁻ anions (Figs. 4a–c) or both TFSI⁻ and PC solvent molecules (Figs. 4d–f). In the absence of MOFs (unactivated condition), Li⁺ showed strong interactions with surrounding species, with binding energies of –5.689 eV for the Li⁺–TFSI⁻ pair in bulk LiTFSI (Fig. 4a, g), and –6.932 eV for Li⁺–TFSI⁻–PC complexes in bulk liquid electrolyte (Fig. 4d, g). Interestingly, when these species were confined within MOF pores, their binding energies significantly decreased (Fig. 4g), indicating that MOFs effectively weaken Li^+^-TFSI⁻ and Li^+^-PC interactions, thereby facilitating Li^+^ transport within the pores compared to bulk conditions. This effect is attributed to specific interactions between MOFs, particularly open metal sites, and TFSI⁻ (Figs. S6c-e) or PC (Figs. S6f, g). Simulation data also revealed that the Li^+^-TFSI⁻ binding energy within the pores of ZIF-8 (-5.302 eV) was relatively higher than that in HKUST-1 (-4.705 eV) (Figs. 4a, b, g). This finding suggests that Li^+^ ions interact more strongly with TFSI⁻ within the confined space of ZIF-8, leading to the formation of a de-solvated, crystal-like Li-TFSI structure, as discussed in Fig. 2c. In addition, weaker binding energy between TFSI⁻ and ZIF-8 compared to HKUST-1 was observed (Figs. S6c-e), likely due to hydrogen shielding effects near Zn²⁺ sites. This further enhances the interactions between Li^+^ and TFSI⁻ within ZIF-8 pores. Upon introducing PC solvent molecules, the total binding energy of Li^+^ within MOF pores increased. Specifically, the Li^+^ binding energy in the HKUST-1 pore increased by more than 20% (from -4.705 to -5.655 eV), whereas in the ZIF-8 pore, it increased by only 18% (from -5.302 to -6.274 eV). This indicates a stronger affinity of PC for Li^+^ in HKUST-1 pores than in ZIF-8 pores, promoting the formation of a solvated Li^+^ structure in the larger pores of HKUST-1. Furthermore, simulations of individual PC solvent molecules within MOF pores revealed stronger PC-MOF interactions in ZIF-8 than in HKUST-1 (Figs. S6f, g). This not only facilitates Li^+^ de-solvation within the small pores of ZIF-8 but also prevents solvent leakage from the porous structure. As a result, ZIF-8-based QSSEs exhibit improved thermal stability but reduced electrode wettability. Conversely, QSSEs based on HKUST-1, with a predominantly solvated Li^+^ structure in its large pores and weaker PC-MOF interactions, offer better electrode wettability, thereby reducing electrolyte/electrode interfacial resistance at the cost of lower thermal stability. Based on these binding energy simulations, detailed solvation and de-solvation structures of Li^+^ in different MOF pore architectures are illustrated in Fig. 4h.


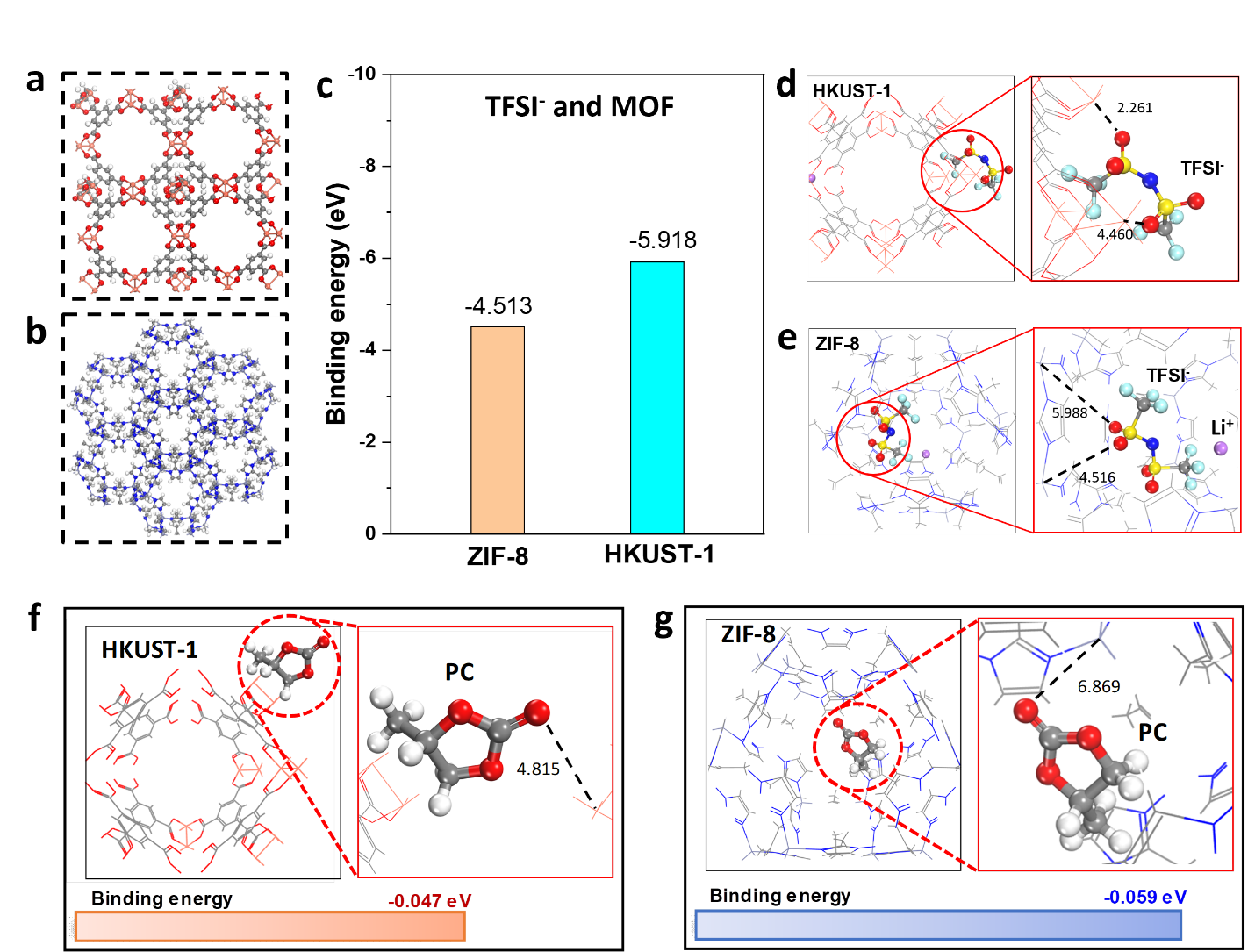


**Fig. S6 a, b** Cluster structures of HKUST-1 (**a**) and ZIF-8 (**b**). **c**, Binding energy of TFSI^−^ anion with different MOFs. **d**,**e**, Simulation snapshots of TFSI⁻ and Li^+^ ions in the pore of HKUST-1 (**d**) and ZIF-8 (**e**). **f**,**g**, Simulation snapshots and binding energy of confined PC molecule in the pore of HKUST-1 (**f**) and ZIF-8 (**g**)

**Note S8 MD simulations of ionic dynamics in 1D MOF-based QSSEs**

To investigate the solvation structures and ion transport behavior of Li⁺ ions within the pores of HKUST-1 and ZIF-8 at the atomistic level, we further performed advanced molecular dynamics (MD) simulations. Our MD simulations employed the Preferred Potential (PFP), a universal neural network potential (UNNP) trained on an extensive DFT dataset covering 96 elemental combinations [S18]. Built upon a deep learning architecture, this potential enables accurate modeling of complex interactions between inorganic metal nodes and organic ligands, offering excellent generalizability and precision across chemically diverse systems [S19]. Such interactions are often inadequately described by conventional classical force fields such as EAM [S20] or the Tersoff potential [S21]. Although reactive models like ReaxFF [S22] are capable of simulating bond formation and breaking, their application to complex systems often requires high computational cost and extensive system-specific parameter tuning. This approach is particularly valuable in metal–organic frameworks (MOFs), where the diversity of coordination environments and complex electrostatics pose a significant challenge for predefined empirical potentials [S23]. By leveraging UNNP, we ensured that subtle interactions, including polarization and metal–solvent affinities, were faithfully captured at near-DFT accuracy [S19]. As such, this simulation protocol represents the most eliable approximation available to connect first-principles energetics with mesoscale dynamics, thereby closely reflecting the real behavior observed in experiments.

The snapshots of the simulated systems in Fig. 4i exhibit marked differences in the local environment of Li⁺ in the two MOFs. In HKUST-1, the large cavities and open Cu²⁺ sites facilitate deep penetration of PC molecules, which exhibit a strong affinity for the framework through favorable electrostatic and coordinative interactions. Consequently, Li⁺ is stabilized in a solvated configuration and experiences only weak perturbation from the framework. In contrast, ZIF-8’s smaller pore window significantly impedes solvent accessibility. In addition, ZIF-8 shows a stronger tendency toward Li⁺–TFSI⁻ pairing and reduced solvation due to its more confined geometry and stronger electrostatic field, consistent with the formation of a de-solvated, crystal-like Li-TFSI complex predicted from DFT results. As a result, Li⁺ in ZIF-8 predominantly adopts a desolvated coordination state. This desolvated state reduces the energy barrier associated with solvation shell reorganization during ion migration and supports a more efficient site-to-site hopping mechanism.

Quantitatively, Li⁺ exhibited significantly higher mobility in ZIF-8 (1.25 × 10⁻⁷ cm² s⁻¹) than in HKUST-1 (4.98 × 10⁻⁸ cm² s⁻¹) (Fig. 4i), despite the more confined pore structure in ZIF-8. This trend aligns well with the ⁷Li MAS NMR results (Figs. 3c,d), where shorter spin-lattice relaxation times in ZIF-8 reflect enhanced local Li⁺ dynamics. This enhanced Li⁺ mobility in ZIF-8 is a direct consequence of the nano-confinement-induced de-solvation effect, wherein Li⁺ ions are stripped of part of their solvation shell upon entry into the sub-nanometer MOF pores, reducing the drag from coordinated solvent molecules and facilitating directional hopping transport along the MOF channels.

In contrast, TFSI⁻ anions exhibited dramatically lower mobility in both MOFs, 1.16 × 10⁻⁸ cm² s⁻¹ in ZIF-8 and 2.97 × 10⁻⁸ cm² s⁻¹ in HKUST-1, as revealed by the near-plateau behavior in their mean square displacement (MSD) curves. However, the mechanisms responsible for this suppression differ fundamentally between the two frameworks. In ZIF-8, the physical size exclusion effect dominates: the narrow pore apertures impose severe steric hindrance on the bulky TFSI⁻ anions. This physical confinement limits the spatial freedom of TFSI⁻ and thereby hinders its contribution to ionic transport. In HKUST-1, although the pores are considerably larger, the presence of open Cu²⁺ coordination sites facilitates strong Lewis acid-base interactions with the electron-rich sulfonyl oxygen atoms of TFSI⁻. These chemical interactions anchor the anions within the framework, reducing their diffusivity through coordination-induced immobilization.

The combined effects of these confinement mechanisms were reflected in the experimentally measured lithium-ion transference numbers ($t_{{Li}^{+}}$, which quantify the fraction of total ionic current carried by Li⁺. Compared to the bulk liquid electrolyte ($t_{{Li}^{+}}$= 0.154), both GHF@HKUST-1 and GHF@ZIF-8 showed substantial improvements, reaching 0.36 and 0.57, respectively (Fig. S7). The much higher $t_{{Li}^{+}}$observed in GHF@ZIF-8 can be attributed to two synergistic factors: (i) the pronounced de-solvation of Li⁺ under spatial confinement, which reduces the ion’s hydrodynamic radius and facilitates faster, directionally selective diffusion through the MOF nanopores; and (ii) the nearly complete exclusion of TFSI⁻ transport due to strong physical sieving, which eliminates the competitive anion migration pathway. In contrast, although HKUST-1 also restricts TFSI⁻ mobility via chemical interactions, its larger pores do not impose the same level of physical confinement, and thus Li⁺ mobility remains relatively lower.

These findings underscore the critical role of nano-confinement architecture, not only in suppressing anion mobility, but also in restructuring the solvation environment of Li⁺ to promote selective and fast ion transport. They further highlight the potential of tailored MOF topologies to overcome fundamental limitations of conventional electrolyte systems and achieve highly directional, cation-dominated ionic conduction in quasi-solid-state architectures.

**Fig. S7 a-c,** Nyquist plots of the Li//Li symmetric cell of GHF@ZIF-8 (**a**), GHF@HKUST-1 QSSE (**b**), and glass fiber (**c**) before and after DC polarization (the insets are the current–time curves at 10 mV polarization)

**Note S9 Influence of pore size and metal–ion interactions on Li^+^ solvation behavior in HKUST-1 and ZIF-8**

The two MOFs impose very different solvation environments because of their pore dimensions and framework chemistries. The wide channels of HKUST-1 are large enough to accommodate intact solvation shells, so Li⁺ tends to migrate in a fully solvated state coordinated with PC. In contrast, the sub-nanometer apertures of ZIF-8 are close to (or smaller than) the size of a solvated Li⁺, which forces partial desolvation as ions pass through the pores. This structural restriction increases the frequency of Li⁺–TFSI⁻ contact and promotes selective ion transport. In terms of framework chemistry, HKUST-1 contains open Cu²⁺ sites that serve as Lewis acidic centers. These sites provide direct coordination pathways with both the sulfonyl oxygen atoms of TFSI⁻ and the carbonyl groups of PC. Such coordination is absent in ZIF-8, where anion and solvent confinement arises primarily from steric sieving and reduced dielectric screening within the narrow apertures. As a result, Li⁺–solvent coordination and metal–anion binding are stronger in HKUST-1, while Li⁺–anion association and steric immobilization are more dominant in ZIF-8.

These conclusions are supported by both experimental and computational evidence. Raman spectra and solid-state ⁷Li NMR identify solvated Li⁺ in HKUST-1 and desolvated Li⁺ in ZIF-8, while XRD of activated ZIF-8 reveals a distinct LiTFSI reflection at 20.7°, confirming stronger Li⁺–anion interactions. DFT binding-energy calculations quantify these differences: Li⁺–TFSI⁻ binding is more favorable in smaller pore size of ZIF-8 (–5.302 eV) than in HKUST-1 (–4.705 eV), consistent with enforced desolvation; PC–MOF framework interaction is also stronger in ZIF-8 (–0.059 eV) than in HKUST-1 (–0.047 eV), explaining why solvent leakage is more effectively suppressed in ZIF-8. Importantly, TFSI⁻ shows a stronger affinity to the open Cu²⁺ sites of HKUST-1 (–5.918 eV), which provides an additional pathway for anion immobilization and enhances the Li⁺–PC interaction for the formation of solvation structure. MD simulations further confirm that Li⁺ diffusivity is higher in ZIF-8 (1.25 × 10⁻⁷ cm² s⁻¹) than in HKUST-1 (4.98 × 10⁻⁸ cm² s⁻¹), while TFSI⁻ mobility is more suppressed in ZIF-8 (1.16 × 10⁻⁸ cm² s⁻¹ vs. 2.97 × 10⁻⁸ cm² s⁻¹ in HKUST-1). These computational results are fully consistent with the experimental transference numbers, which increase from 0.36 for GHF@HKUST-1 to 0.57 for GHF@ZIF-8. Taken together, the data show that HKUST-1 favors solvated Li⁺ transport, while ZIF-8 enforces desolvation, and immobilizes both solvent and anions.

**Note S10 Properties of core-shell MOF-based QSSE designed by 1D ZIF-8 and HKUST-1**

To harness the advantages and mitigate the limitations of individual MOFs, we constructed a core–shell structure, GHF@ZIF-8@HKUST-1, by growing HKUST-1 as an outer shell on the pre-formed GHF@ZIF-8. SEM images reveal that the resulting core–shell architecture exhibits near-complete coverage of the surface of each individual fiber as well as the entire fiber network within the GF membrane (Supplementary Figs. 8a-g). EDS elemental mapping further confirms the successful formation of the hierarchical structure: the Zn signal (yellow), representing the ZIF-8 core, is primarily concentrated near the inner region adjacent to the GF (Si, navy), while the Cu signal (sky-blue), indicative of HKUST-1, is clearly located on the outermost surface (Figs. S8c-f). To further verify this structure, FIB cross-sections combined with EDS mapping and spectrum (Fig. S9) clearly show a continuous Cu-rich shell uniformly covering the Zn-rich core. High-resolution TEM with EDS analysis (Fig. S10) further supports this result, revealing at higher magnification the distinct spatial separation between the Zn-rich inner domain and the Cu-rich outer layer with a well-defined, seamless interface. The XRD pattern of GHF@ZIF-8@HKUST-1 exhibits characteristic diffraction peaks corresponding to both ZIF-8 and HKUST-1 when compared to their standard references, corroborating the formation of the ZIF-8@HKUST-1 core–shell MOF structure on the GHF substrate (Fig. S8h). Following our two-step activation process with a typical liquid electrolyte (1 M LiTFSI in PC), the intensity of the XRD peaks, especially in the low 2θ region (5–10°), was significantly reduced (Fig. S8h). This reduction in peak intensity indicates that the electrolyte molecules were effectively infiltrated into both the HKUST-1 shell and the ZIF-8 core during activation. The infiltration likely disrupted the long-range ordering of the framework structure, leading to diminished XRD signal intensity associated with the crystallographic planes of the MOF layers.


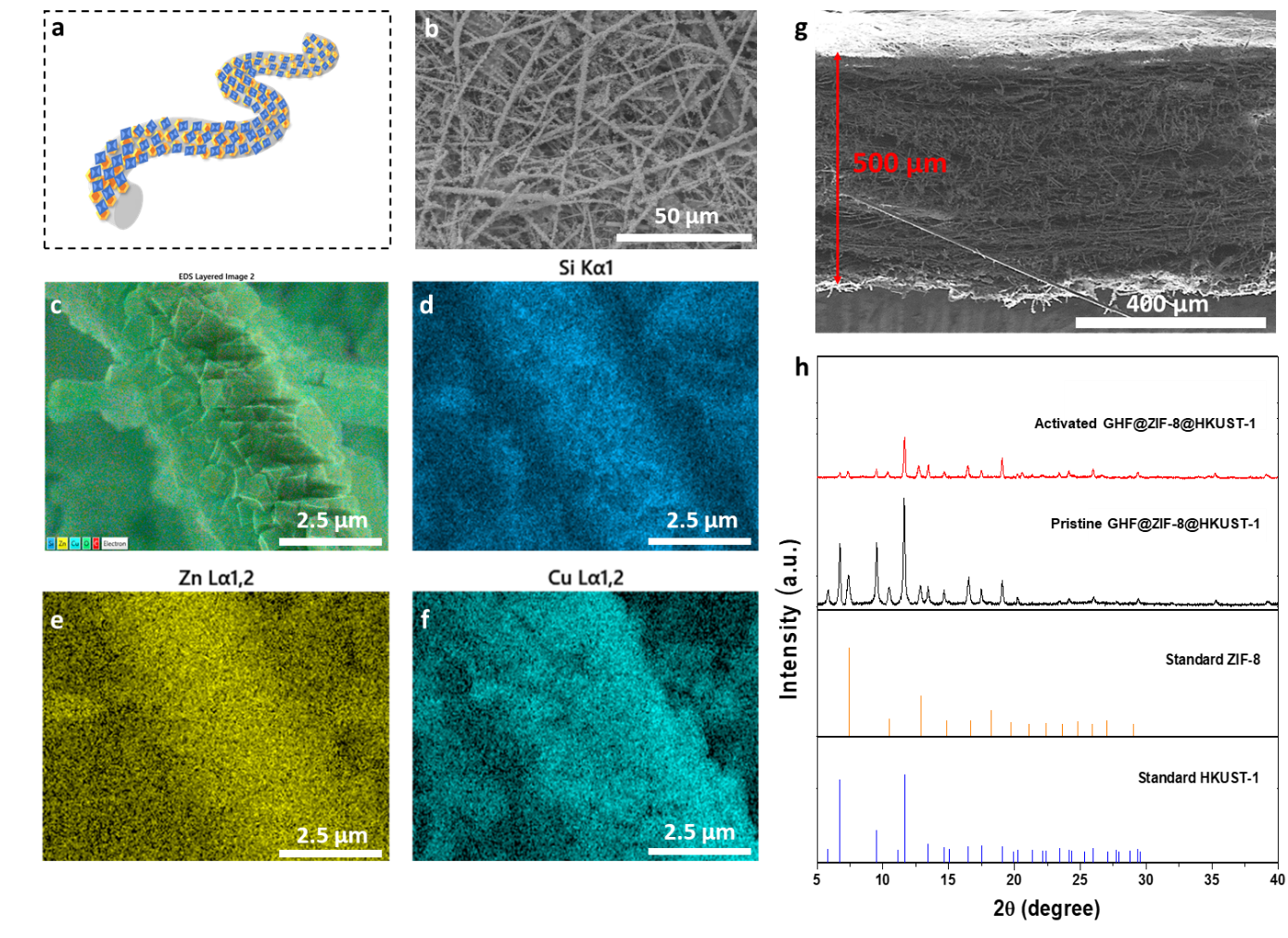


**Fig. S8 a,** Schematic illustration of ZIF-8@HKUST-1 core-shell grown on GHF. **b**, Low magnification SEM images of ZIF-8@HKUST-1 core-shell grown on GHF. **c-f,** High magnification SEM images of ZIF-8@HKUST-1 core-shell grown on single GHF with EDS layer (**c**) and elemental mapping images of GHF@ZIF-8@HKUST-1 (**d-f**). **g**, Cross-sectional SEM image of GHF@ZIF-8@HKUST-1 quasi-solid-state electrolyte. **h**, XRD patterns of pristine and activated GHF@ZIF-8@HKUST-1


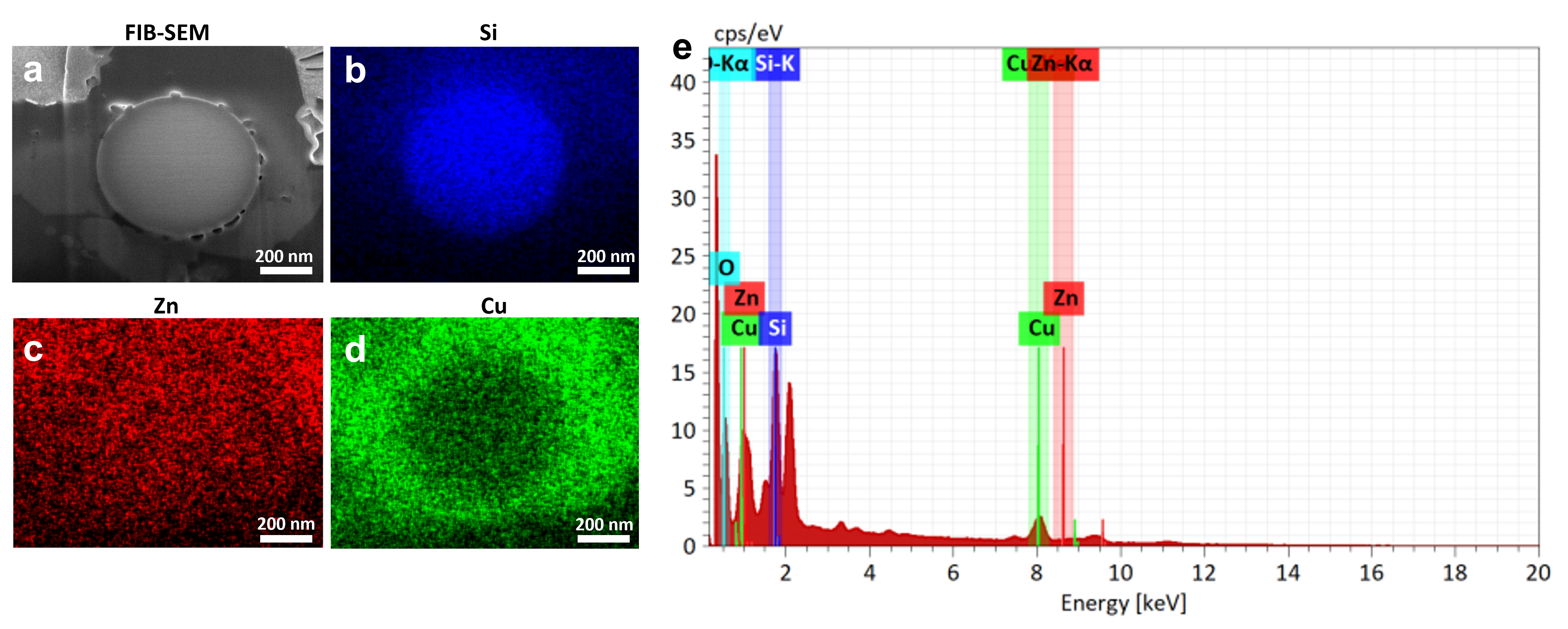


**Fig. S9 a-e,** FIB cross-sectional images of ZIF-8@HKUST-1 core-shell grown on single GHF **(a)** with elemental mapping images **(b-d)** and EDS spectrum **(e)** of GHF@ZIF-8@HKUST-1


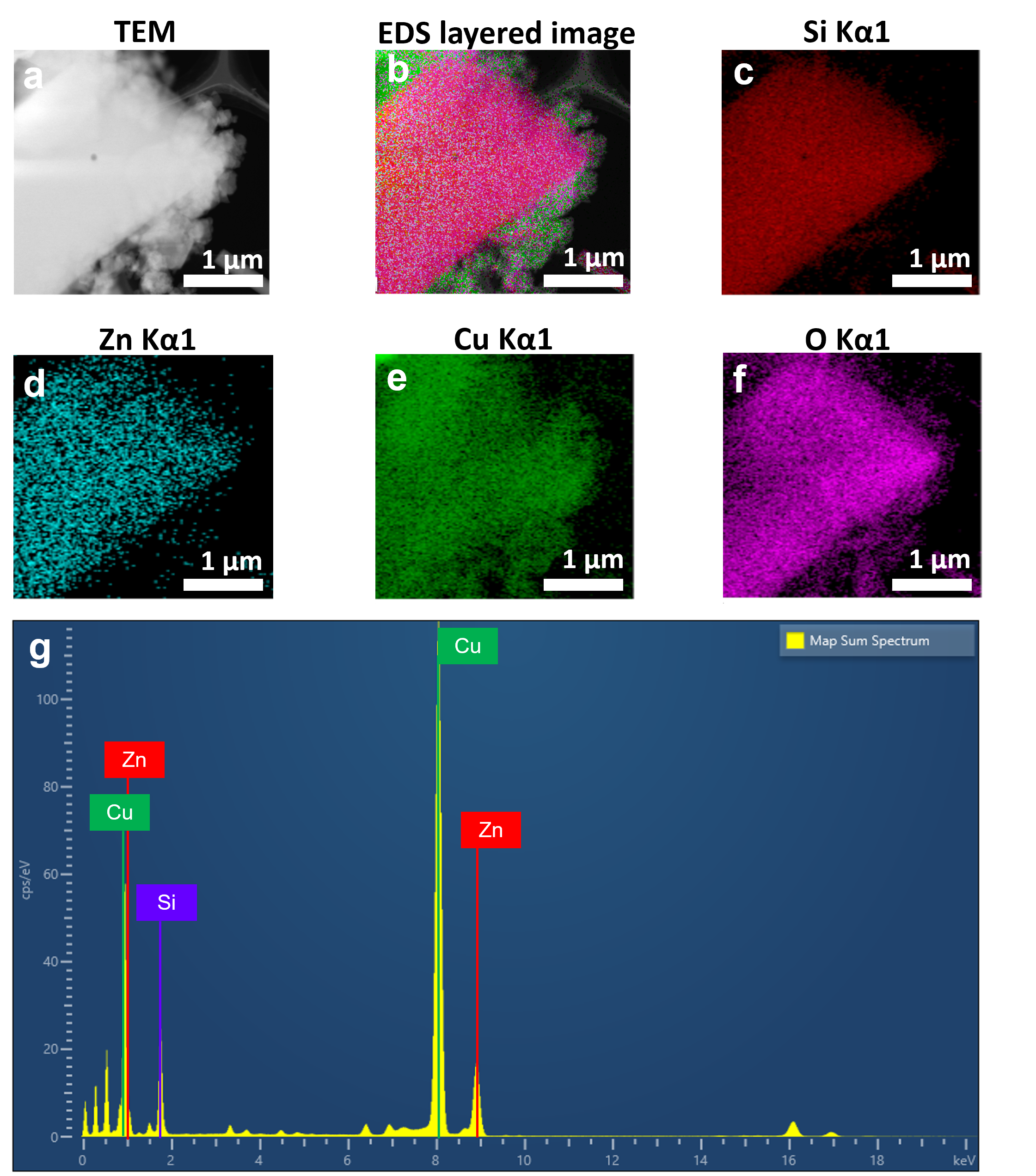


**Fig. S10 a-f,** TEM images of ZIF-8@HKUST-1 core-shell grown on single GHF **(a)** with EDS layer **(b)**, elemental mapping images **(c-f)**, and EDS spectrum **(g)** of GHF@ZIF-8@HKUST-1

**Note S11 Thermal stability of core-shell MOF-based QSSE designed by 1D ZIF-8 and HKUST-1**

To assess thermal stability under operating conditions, TGA coupled with GC analysis was performed for two representative samples: (i) the GHF@ZIF-8@HKUST-1 QSSE and (ii) a conventional liquid electrolyte consisting of 1 M LiTFSI in PC (Fig. S11). Both were held at 100 °C for 2 h. The QSSE exhibited only a slight weight loss of 0.89%, and GC analysis identified the released gas mainly as O_2_ (Figs. S11b, e), which we attribute to pre-adsorbed species in the pores rather than solvent evaporation. No signals from PC were detected, indicating that solvent molecules confined within the MOF channels are strongly stabilized. By contrast, the liquid electrolyte showed a dramatic 35.67% weight loss, and GC analysis confirmed that the evolved gas was PC vapor (Figs. S11d, f), directly evidencing the high volatility of free solvent. **To further validate these results, we extended TGA–GC measurements up to 150 °C (Fig. S12). In this broader temperature range, bulk LE began releasing PC gas at relatively low temperatures, and the amount of PC vapor increased sharply as the temperature rose (Figs. S12d, f, g). At around 150 °C, additional decomposition gases such as CO_2_ and CO were also detected (Figs. S12d ,h), confirming severe thermal decomposition of the free electrolyte. In contrast, the GHF@ZIF-8@HKUST-1 QSSE delayed the onset of PC gas release until nearly 150 °C, and even then, the amount was negligible with no CO_2_/CO signals observed (Figs. S12b, e).** This stark difference highlights the stabilizing effect of MOF confinement: solvent molecules immobilized in well-defined pores experience steric restriction and host–guest interactions that suppress evaporation, whereas free liquid solvent rapidly escapes at elevated temperature. Such thermal robustness of the QSSE explains its ability to maintain structure and composition, thereby supporting stable electrochemical cycling even under harsh conditions. The ability of the MOF-based QSSE to suppress not only solvent loss but also secondary gas formation provides direct evidence for its enhanced safety and long-term reliability under high-temperature conditions.


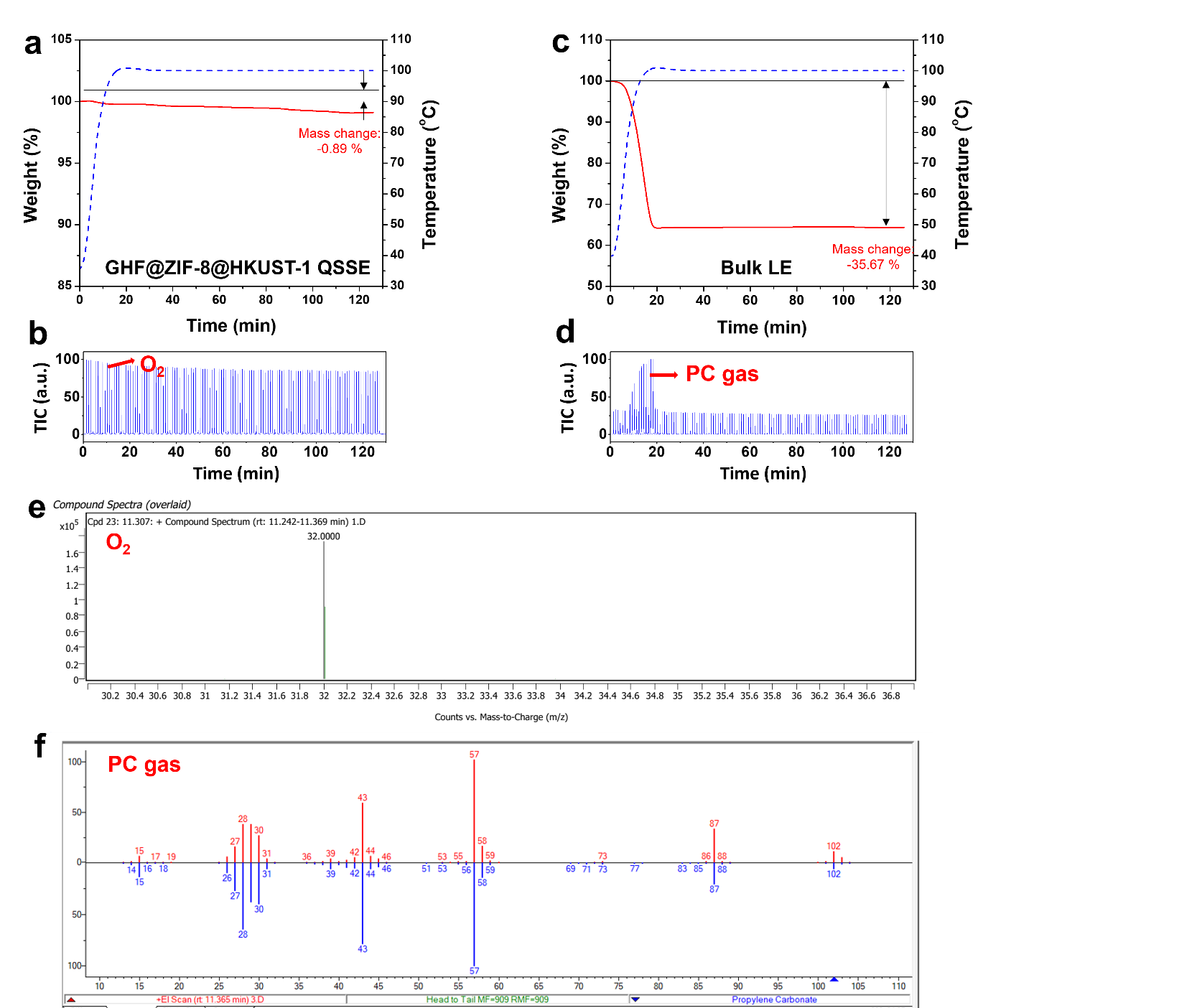


**Fig. S11 a, b,** Thermogravimetric analysis (TGA) curves of GHF@ZIF-8@HKUST-1 QSSE (**a**) and bulk LE (**b**). **c,d,** Total Ion Chromatograms (TIC) graphs of gas generated from GHF@ZIF-8@HKUST-1 QSSE (**c**) and bulk LE (**d**). **e, f,** Gas species identified by gas chromatography–mass spectrometry (GC–MS) for GHF@ZIF-8@HKUST-1 QSSE (**e**) and bulk LE (**f**). The sample temperature was increased from room temperature to 100 °C at a heating rate of 10 °C min⁻¹ and then maintained at 100 °C for 2 h


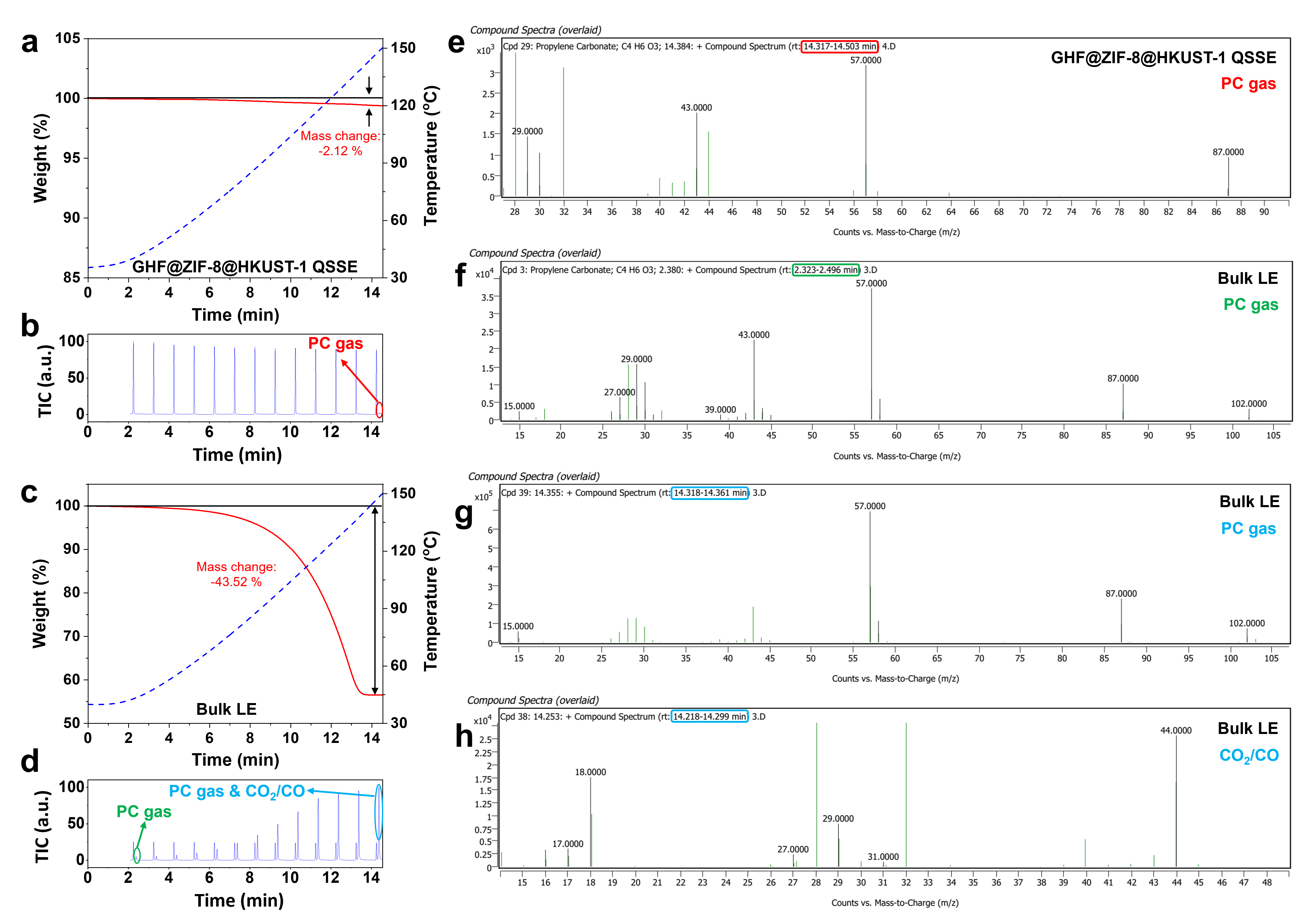


**Fig. S12 a, c,** Thermogravimetric analysis (TGA) curves of GHF@ZIF-8@HKUST-1 QSSE (**a**) and bulk LE (**c**). **b,d,** Total Ion Chromatograms (TIC) graphs of gas generated from GHF@ZIF-8@HKUST-1 QSSE (**b**) and bulk LE (**d**). **e-h,** Gas species identified by gas chromatography–mass spectrometry (GC–MS) for GHF@ZIF-8@HKUST-1 QSSE (**e**) and bulk LE (**f-h**). The sample temperature was increased from room temperature to 150 °C at a heating rate of 10 °C min⁻¹


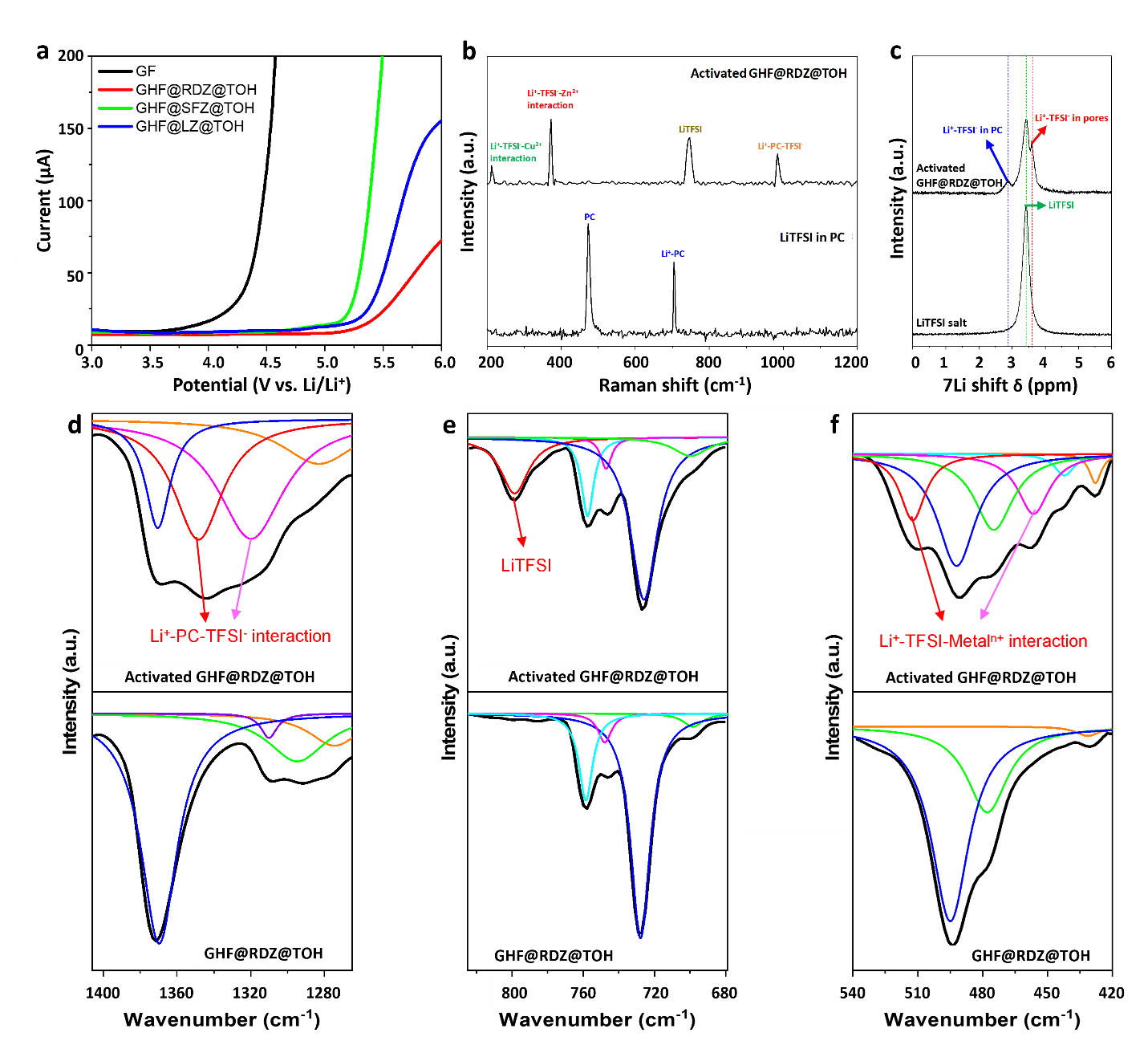


**Fig. S13 a,** Linear sweep voltammetry (LSV) curves of GF and different core-shell MOF-based QSSEs. **b**, Raman spectra of LiTFSI in PC and GHF@ZIF-8@HKUST-1 (GHF@RDZ@TOH) QSSE. **c-e,** Fourier transform infrared spectra (FT-IR) of pristine and activated GHF@ZIF-8@HKUST-1 (GHF@RDZ@TOH) separators in high (**c**), middle (**d**), and low (**e**) wavenumber ranges

**
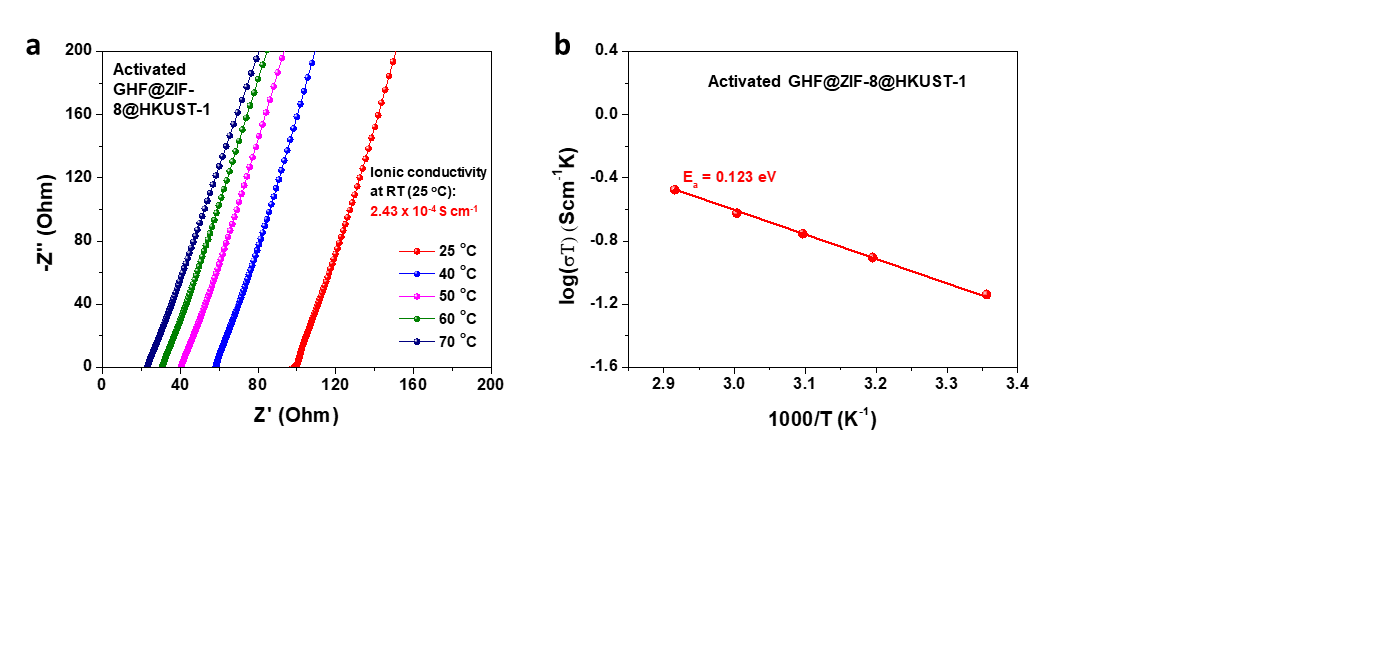
**

**Fig. S14 a, b,** Nyquist plots of activated GHF@ZIF-8@HKUST-1 at various temperatures from 25 to 70 ^o^C (**a**) and its corresponding Arrhenius plot (**b**)

**Note S12 The effects of lattice mismatch between ZIF-8 core and HKUST-1 cell on Li⁺ and TFSI⁻ transport**

Our extended analysis of the crystal structure of core-shell shows significant lattice distortion in both ZIF-8 and HKUST-1 at their surface contact region, leading to the formation of a lattice mismatch region between ZIF-8 and HKUST-1. To gain deeper insight into the structural evolution at the MOF–MOF interface, we compared the characteristic diffraction peaks of ZIF-8 and HKUST-1 in the core–shell architecture with standard XRD patterns of ZIF-8 and HKUST-1, respectively. Notably, the (111) and (200) peaks of HKUST-1 exhibited slight shifts toward higher 2θ angles, while the (110) peak of HKUST-1 shifted toward lower 2θ (Fig. S15). These peak shifts suggest lattice distortion at the ZIF-8/HKUST-1 interface, likely caused by crystallographic mismatch during the HKUST-1 growth on the ZIF-8 core. The mismatch results in local compression of HKUST-1 and expansion of ZIF-8 near the interface, leading to a strained and disordered interfacial region.

This region plays a critical role in influencing the transport behaviors of Li⁺ and TFSI⁻ ions (Fig. S16 and Table S2). Specifically, the structural expansion and strain-induced defects slightly widen the Li⁺ diffusion channels, lowering the activation energy required for ion migration. While the resulting misalignment of pore networks can hinder ion transport, the small size of Li⁺ (~0.64 Å) [24] allows it to bypass steric constraints through site-to-site hopping and defect-mediated conduction pathways. In contrast, the much larger TFSI⁻ anions face severe steric hindrance and reduced mobility due to pore misalignment. Furthermore, defect-induced charge redistribution introduces localized positive sites (e.g., Cu²⁺ in HKUST-1 and Zn²⁺ in ZIF-8), which serve as Lewis acid sites that preferentially coordinate with Li⁺, further promoting its mobility. Simultaneously, TFSI⁻ is more likely to be trapped at defect sites via electrostatic interactions. The contrast in surface polarity, hydrophobic ZIF-8 versus hydrophilic HKUST-1, also contributes to this transport selectivity. While Li⁺ can readily diffuse through both domains, the disruption in hydrophilic environments compromises TFSI⁻ solvation and impedes its transport.

Collectively, the lattice mismatch region serves as a unique, third Li⁺ conduction pathway, beyond the individual MOF channels, by facilitating Li⁺ migration while suppressing TFSI⁻ diffusion. This asymmetric ion transport behavior contributes to the superior Li⁺ conductivity and electrochemical performance of the GHF@ZIF-8@HKUST-1-based QSSE. Future studies involving advanced simulations and spectroscopic characterization will further elucidate the mechanistic role of the interfacial lattice mismatch in governing ion transport dynamics.

**
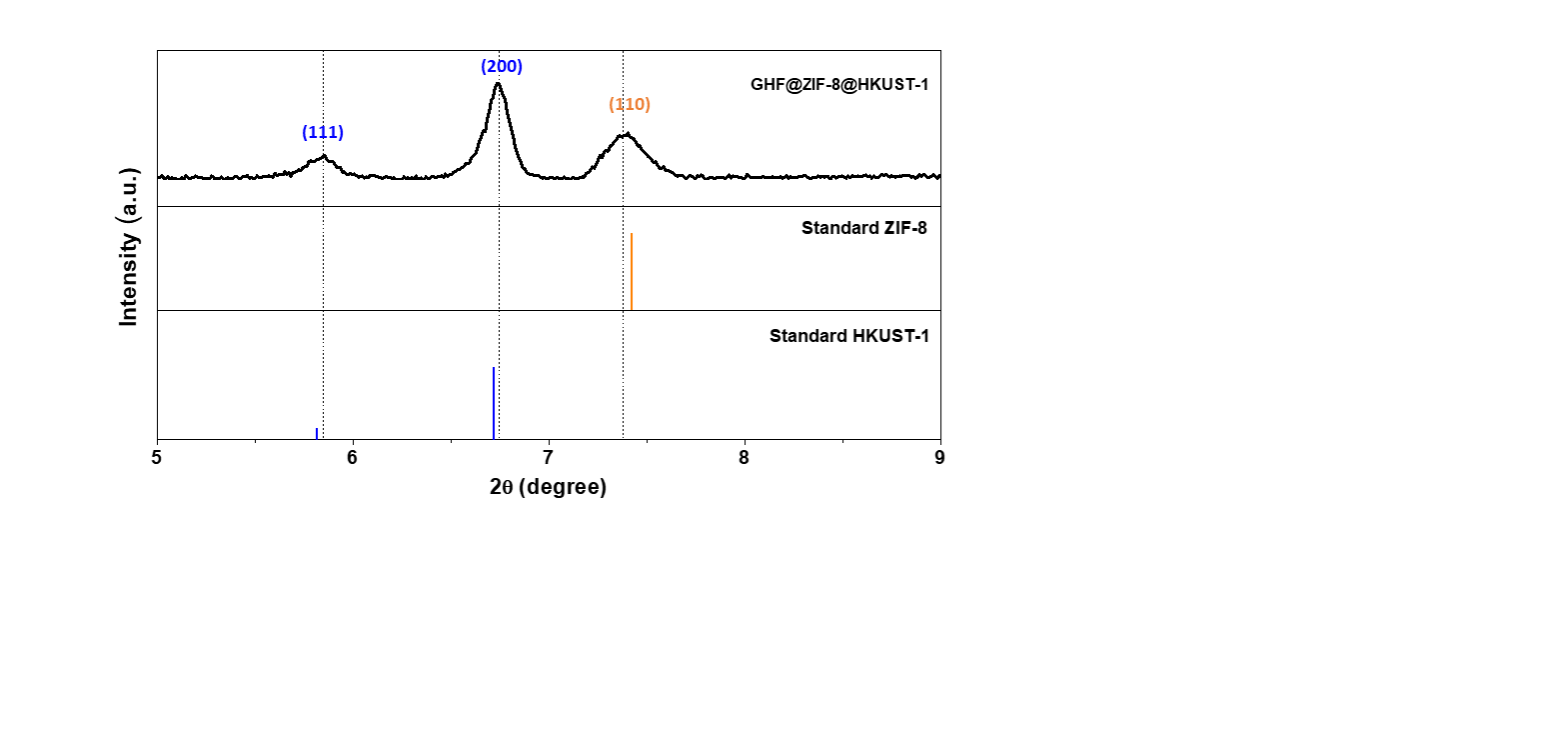
**

**Fig. S15** Peak shifting in XRD pattern of GHF@ZIF-8@HKUST-1 core-shell

**
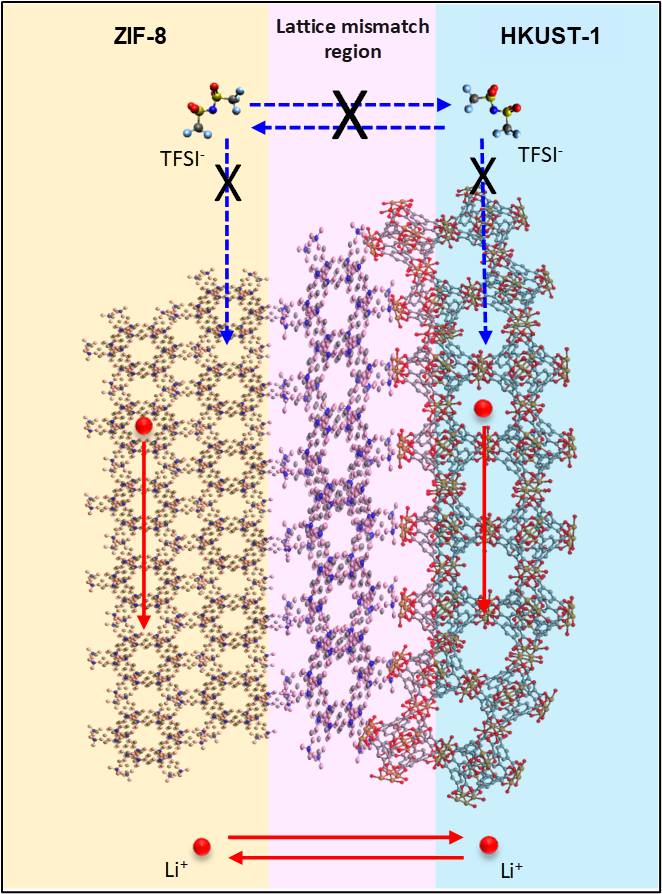
**

**Fig. S16** Schematic illustrations of ion transport between the two main ionic conduction channels, either enhanced or restricted by the large lattice mismatch between the core (ZIF-8) and shell (HKUST-1) materials

**Table S2** Summary of the key effects of large lattice mismatch on Li⁺ and TFSI⁻ transport

| **Factor** | **Effect on Li⁺ Transport (Enhanced)** | **Effect on TFSI⁻ Transport (Suppressed)** |
| --- | --- | --- |
| **1. Structural Expansion & Localized Disorder** | **Localized strain may expand diffusion channels slightly**, lowering the activation energy for Li⁺ diffusion. | **Distorted structures** create misaligned pore channels, making it **difficult for TFSI⁻ to migrate.** |
| **2. Pore Connectivity** | Some **misaligned pores may still allow Li⁺ hopping** between ZIF-8 and HKUST-1.  **Small size of Li⁺ (~1.8 Å in bare form, ~3-4 Å in solvated form)** allows it to move through defects and narrow pathways. | **Large TFSI⁻ anions get sterically blocked** by constricted or misaligned pores. |
| **3. Charge Distribution & Defect Trapping** | **Defects introduce positive charge sites** (e.g., Cu²⁺ in HKUST-1), which can act as **Li⁺ anchoring points**, promoting rapid Li⁺ transport by enhancing ion hopping between adjacent sites. | TFSI⁻ **may get trapped in defect-rich interfacial zones**, significantly reducing its diffusion. |
| **4. Hydrophobic/Hydrophilic Contrast** | **ZIF-8 is more hydrophobic**, but Li⁺ (when de-solvated) can still diffuse through. | **TFSI⁻ prefers a hydrophilic environment (like HKUST-1), but the interface disrupts this**, making anion transport inefficient. |

**Note S13 The cell performance of Li//Li symmetric cells at room temperature**

Before evaluating the performance of LMBs designed with our QSSEs, we first investigated the behavior of Li plating/stripping through these QSSEs in Li//Li symmetric cells at room temperature (25 °C) with a current density of 1 mA cm^−2^ (areal capacity of 1 mAh cm^−2^) (Fig. S17). For comparison, symmetric cells using commercial GF separators and liquid electrolyte (LE) were also assembled and tested. The results showed unstable voltage polarization and short-circuiting after approximately 100 hours. Replacing the LE and GF separator with QSSEs based on single MOFs grown on GHF significantly improved the Li plating and stripping process, thereby enhancing the performance of the Li//Li symmetric cells. However, these QSSEs still exhibited some limitations over extended operation periods, such as short-circuiting in the cell with GHF@HKUST-1 QSSE after about 260 hours and a sudden increase in voltage polarization in the cell using GHF@ZIF-8 QSSE after 500 hours. Notably, the QSSE based on the ZIF-8@HKUST-1 core-shell structure grown on GHF overcame these limitations, maintaining excellent performance in Li//Li symmetric cells with the ability to sustain long-term cycling stability for over 850 hours without short-circuiting or significant voltage polarization.

**
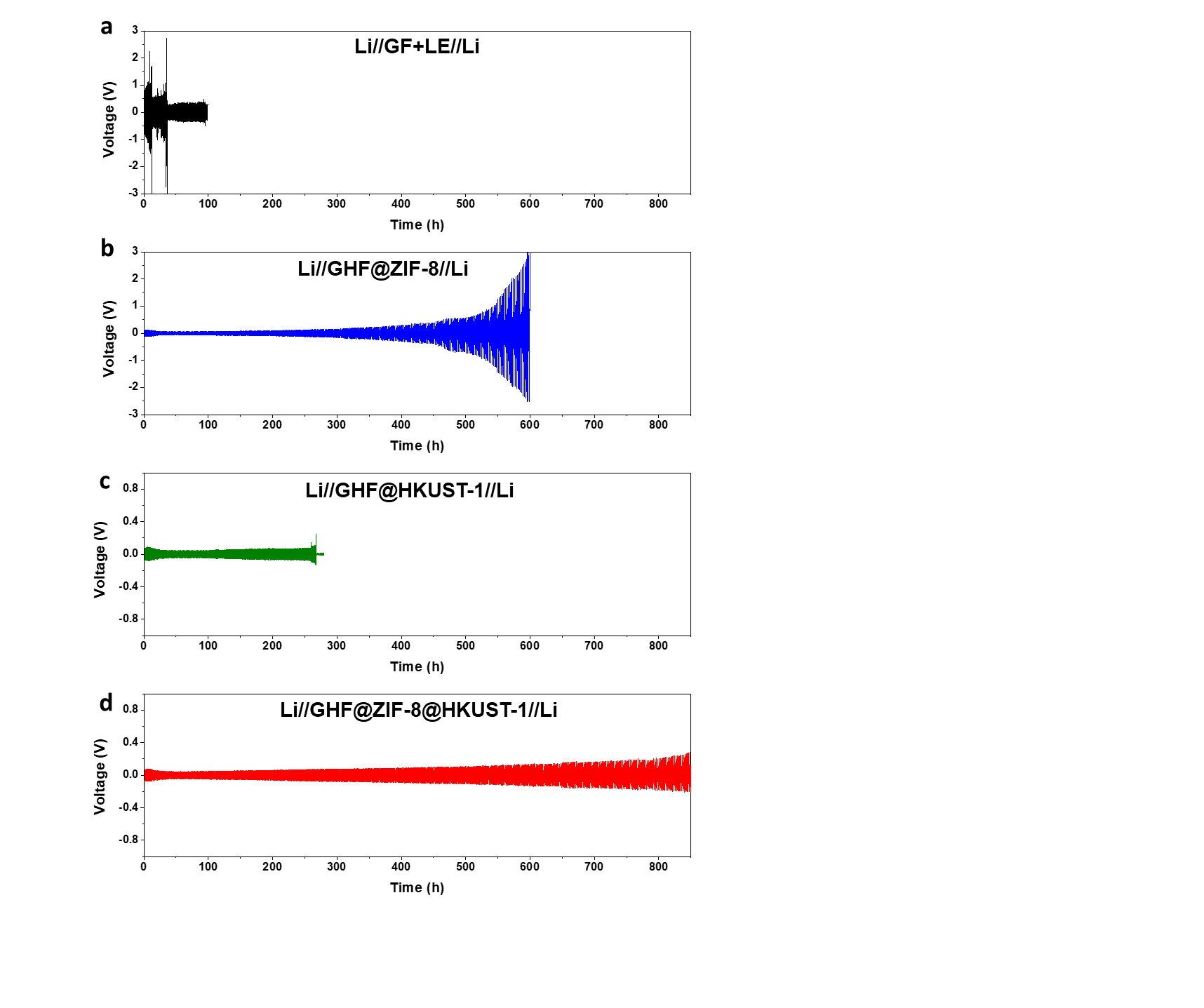
**

**Fig. S17 a-d**, Galvanostatic cycling of Li//Li symmetric cells assembled with conventional GF membrane combined with typical liquid electrolyte (LE) (**a**) and QSSEs based on GHF@ZIF-8 (**b**), GHF@HKUST-1 (**c**) and GHF@ZIF-8@HKUST-1 (**d**) at current densities of 1 mA cm^−2^ at room temperature (RT)

**Note S14 The cell performance of Li//LFP cells at room temperature**

We evaluated Li//LFP cells at room temperature to confirm the intrinsic electrochemical stability of the MOF-based QSSEs under mild operating conditions (Fig. S18). All tested cells maintained stable cycling for 100 cycles with Coulombic efficiencies above 99%. **The GHF@ZIF-8@HKUST-1 QSSE cell delivered a discharge capacity exceeding 149 mAh g^-1^ after 100 cycles, which was 1.16 times higher than that of the control GF+LE cell (approximately 128 mAh g⁻¹), and retained more than 98% of its capacity after 100 cycles.** The GF+LE cell exhibited relatively lower discharge capacity, which we attribute to the formation of a thicker SEI due to strongly solvated Li⁺. This interpretation is further supported by the abnormal polarization evolution observed in Fig. S19, where the overpotential of the GF+LE cell initially decreased but subsequently increased after prolonged cycling, reflecting unstable interfacial chemistry and repeated SEI reconstruction. **Quantitatively, the overpotential (ΔV) of the GF+LE cell exceeded 170 mV after 10 cycles, nearly 1.4 times higher than that of the GHF@ZIF-8@HKUST-1 QSSE (around 120 mV), indicating severe interfacial polarization in the MOF-free system.** In contrast, the MOF-based QSSEs promoted partial desolvation of Li⁺ and the formation of a thinner, more conductive SEI, resulting in improved discharge capacity and interfacial stability. Notably, the GHF@ZIF-8@HKUST-1 QSSE maintained a consistently low and stable polarization throughout cycling, underscoring the effectiveness of MOF confinement in promoting partial Li⁺ desolvation and stabilizing the electrode and electrolyte interface. **Furthermore, EIS measurements of Li//LFP full cells (Fig. S20) revealed a significantly lower charge-transfer resistance (R_ct_ approximately 26 Ω) for the GHF@ZIF-8@HKUST-1 QSSE compared to the GF+LE cell (R_ct_ approximately 42 Ω). This lower resistance demonstrates more favorable Li⁺ migration and interfacial kinetics within the MOF-confined electrolyte, where partial desolvation reduces the energy barrier for charge transfer and prevents the accumulation of resistive interphases during cycling.**


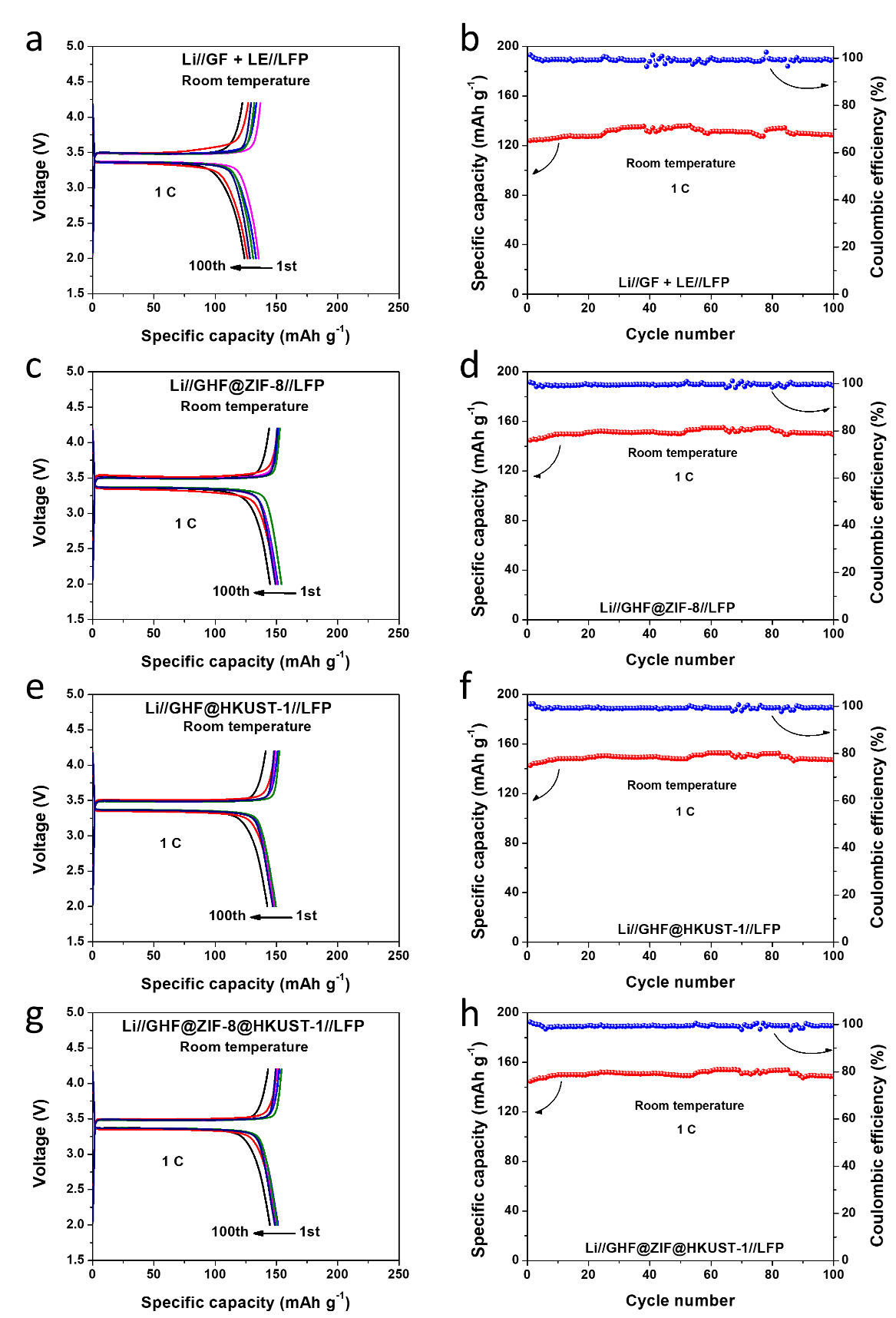


**Fig. S18 a-h**, The cyclic performance of Li//LFP cells at room temperature using: GF + LE (**a, b**), GHF@ZIF-8 (**c, d**), GHF@HKUST-1 (**e, f),** and GHF@ZIF-8@HKUST-1 (**g, h)**


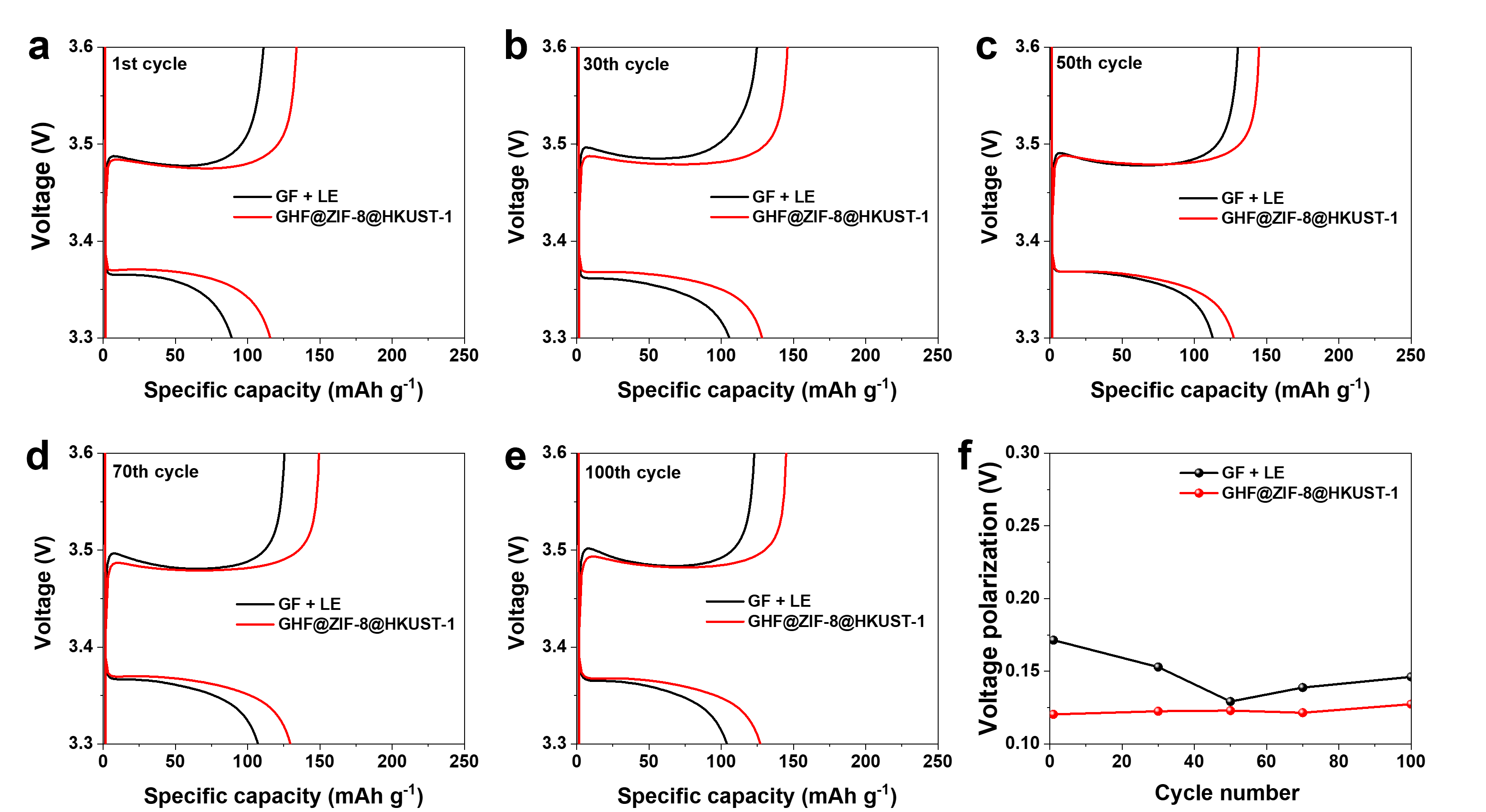


**Fig. S19 a-e**, The galvanostatic charge–discharge (GCD) curves of Li//LFP cells using GF + LE and GHF@ZIF-8@HKUST-1 at room temperature at different cycles**. f,** Comparison of voltage polarization evolution of Li//LFP cells using GF + LE and GHF@ZIF-8@HKUST-1 QSSE over 100 cycles at room temperature

**Fig. S20**  Nyquist plots of Li//LFP full cells using GF + LE and GHF@ZIF-8@HKUST-1 QSSE at room temperature

**Note S15 Contact angle measurement of MOF-based separators**

To validate the influence of interfacial wetting on cell performance, contact angle measurements were performed with the liquid electrolyte on different GHF@MOF membranes (Fig. S21). The GHF@ZIF-8 membrane exhibited a relatively high contact angle (36.7°), indicating poor wettability and limited electrolyte infiltration. In contrast, the GHF@HKUST-1 membrane showed a much lower contact angle (11.7°), confirming enhanced wettability due to its hydrophilic nature and open metal sites. The core–shell GHF@ZIF-8@HKUST-1 membrane displayed an intermediate contact angle (19.1°), demonstrating that the outer HKUST-1 shell significantly improves electrolyte affinity compared to ZIF-8 alone. These results provide direct evidence that improved interfacial wetting contributes to the enhanced cycling stability of MOF-based QSSEs, particularly under high-temperature operation.


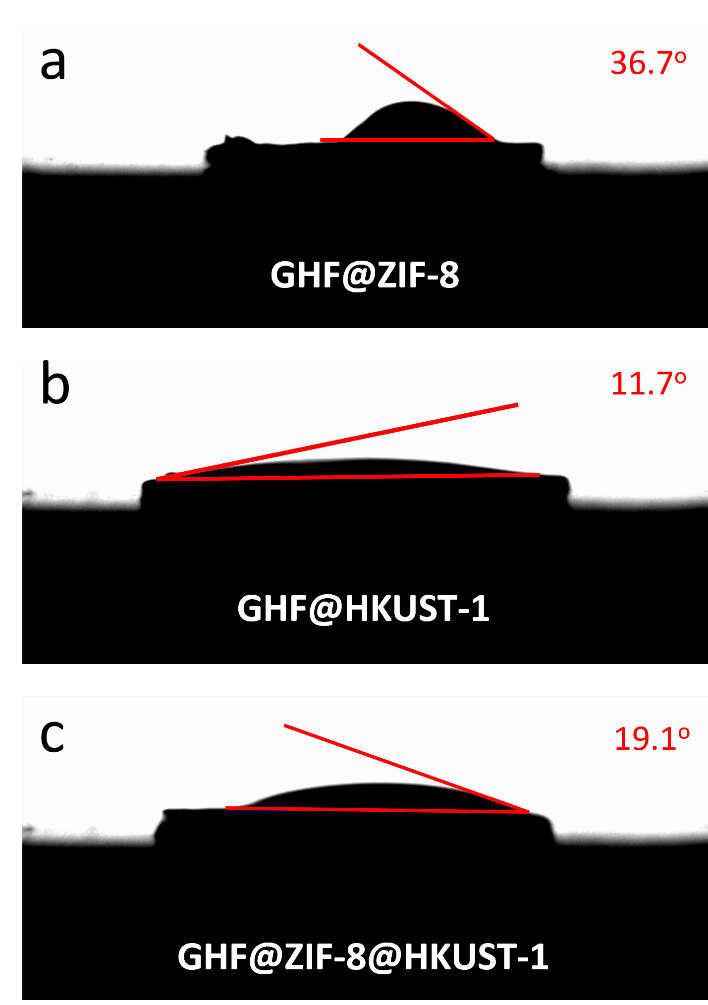


**Fig. S21 a-c,** Contact angle measurement of MOF-based separators: GHF@ZIF-8 **(a)**, GHF@HKUST-1 **(b)**, and GHF@ZIF-8@HKUST-1 **(c)**

**Note S16 Robust SEI layers of core-shell MOF-based QSSE**

To directly analyze the interfacial chemistry and confirm the SEI composition, we performed XPS analyses of Li electrodes retrieved from cells assembled with (i) a GF separator and liquid electrolyte (1 M LiTFSI in PC) and (ii) the GHF@ZIF-8@HKUST-1 QSSE after cycling at 100 °C (Fig. S22). The spectra of pristine Li showed essentially flat baselines without any detectable F 1s, N 1s, S 2p, or carbonate-related C 1s peaks, confirming that the Li surface was clean and free of SEI prior to cycling. After cycling, the GF+LE cell showed a surface dominated by organic decomposition products, with strong C 1s peaks at 284–288 eV (C–H, C–O, C=O) and weak inorganic peaks, indicating that inorganic phases were only a minor fraction (~28.5%). In contrast, the electrode cycled with the GHF@ZIF-8@HKUST-1 QSSE displayed a markedly different profile, characterized by a strong F 1s peak at ~685 eV (LiF), a distinct C 1s contribution near 290 eV (Li_2_CO_3_), and additional inorganic components including Li₃N (N 1s at ~398 eV) and Li₂SO₃ (S 2p at ~168 eV), together accounting for ~78.9% of the SEI. These results clearly demonstrate that the SEI only forms after electrochemical cycling and that the MOF-based QSSE promotes the preferential formation of an inorganic-rich interphase. The confined pores suppress solvent activity and enforce partial Li⁺ desolvation, thereby limiting solvent-driven reduction pathways that generate organic species. Consequently, the interphase formed in the QSSE system is thinner, more inorganic-dominated, and mechanically robust, which directly explains the superior cycling stability of both Li//Li symmetric and Li//LFP cells at elevated temperature.


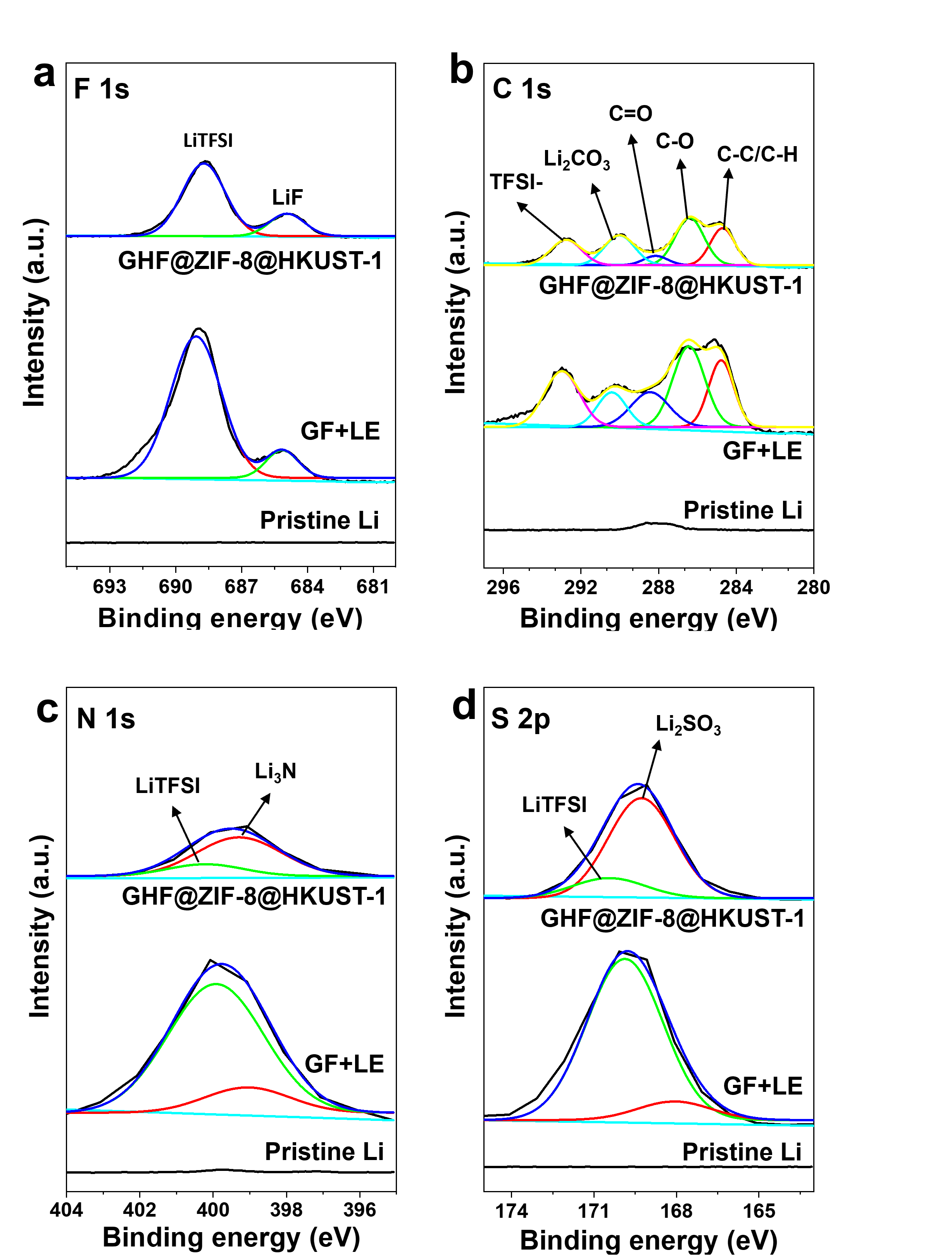


**Fig. S22** XPS spectra of different elements on pristine Li metal surface and Li electrodes detached from Li//LFP cells with GF+LE and GHF@ZIF-8@HKUST-1 QSSE after cycling at 100 ^o^C

**Note S17 The cycling failure mechanism of Li//LFP cells using MOF-based QSSEs**

To gain deeper insights into the cycling failure mechanisms of Li//LFP cells at elevated temperature (100 °C), we conducted post-mortem characterizations of both the Li electrodes and the GHF@MOF membranes after prolonged cycling.

SEM analyses of the cycled Li metal surfaces revealed distinct morphologies depending on the electrolyte used (Fig. S23). In the case of GHF@HKUST-1, the Li surface was covered with dense and disordered dendritic deposits (Figs. S23a, d), consistent with short-circuit failure during cycling. This is attributed to the migration of solvated Li⁺ ions through the large pores of HKUST-1, where uneven desolvation at the electrode interface generated localized current hot spots and promoted dendrite growth. In contrast, cells employing GHF@ZIF-8 displayed smoother Li surfaces with limited dendrite formation (Figs. S23b, e), reflecting the beneficial effect of partial Li⁺ desolvation in the narrow ZIF-8 pores. However, the strong confinement of PC solvent in ZIF-8 reduced interfacial wettability and eventually caused electrolyte depletion, leading to increased polarization and rapid capacity fading.

For the GHF@ZIF-8@HKUST-1 electrolyte, the Li surface remained smooth and uniform even after extended cycling at 100 °C (Figs. S23c, f). This superior morphology originates from the synergistic dual-channel transport mechanism: the inner ZIF-8 channels induce partial desolvation and promote uniform Li deposition, while the outer HKUST-1 shell ensures sufficient solvent supply and good electrode wetting. As a result, the core–shell QSSE effectively suppresses dendrite growth and electrolyte depletion, enabling long-term cycling stability.

In addition, stability analysis of the membranes confirmed that all MOF-based QSSEs retained their structural integrity after cycling, with no observable degradation or collapse of the frameworks (Fig. S24). This indicates that the performance differences among the three systems primarily arise from interfacial ion transport behaviors rather than membrane instability.


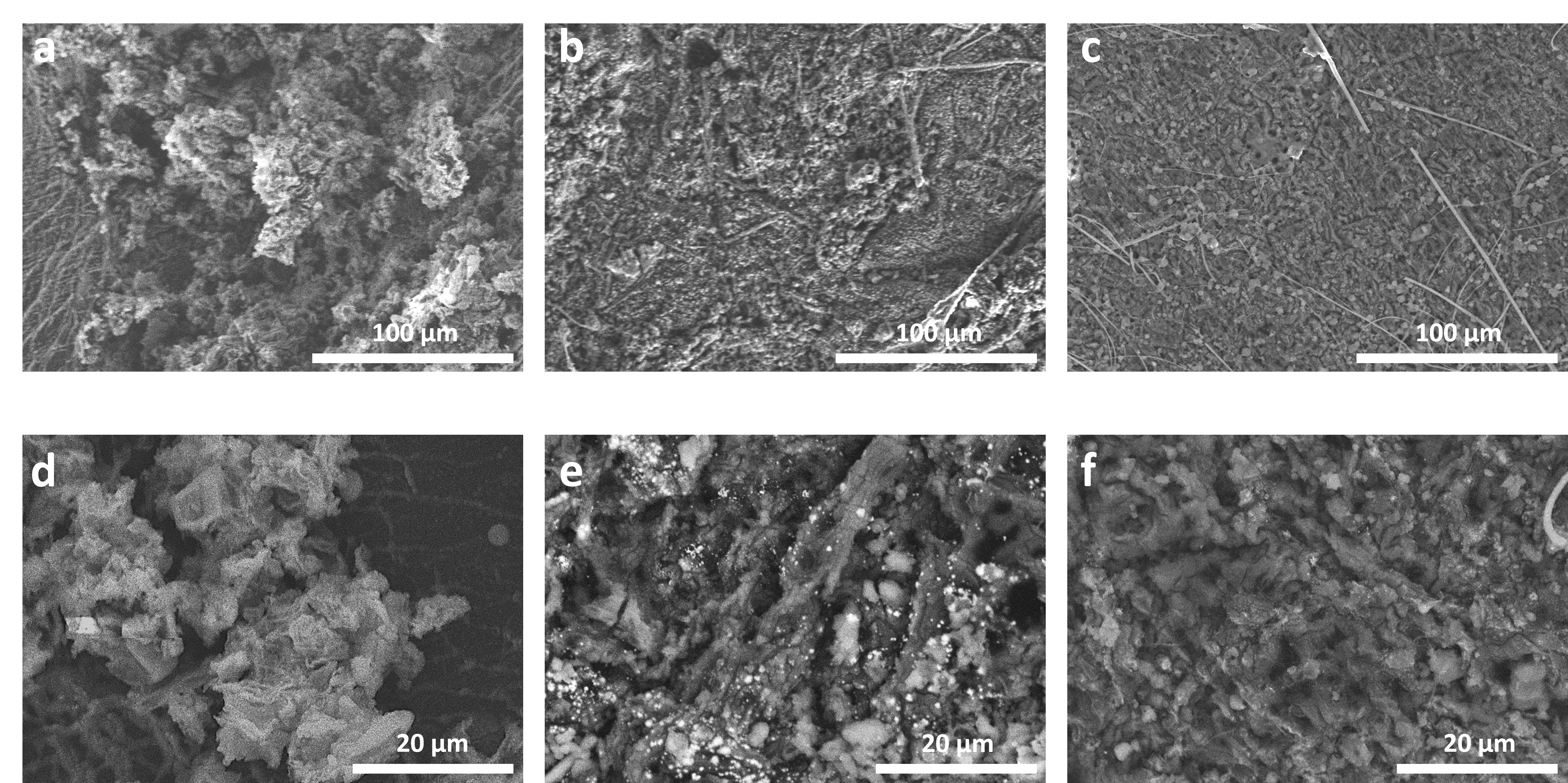


**Fig. S23** **a-f,** SEM images of Li metal surfaces detached from the Li//LFP cell using GHF@ZIF-8 (**a, d**), GHF@HKUST-1 (**b, e**) and GHF@ZIF-8@HKUST-1 (**c, f**)


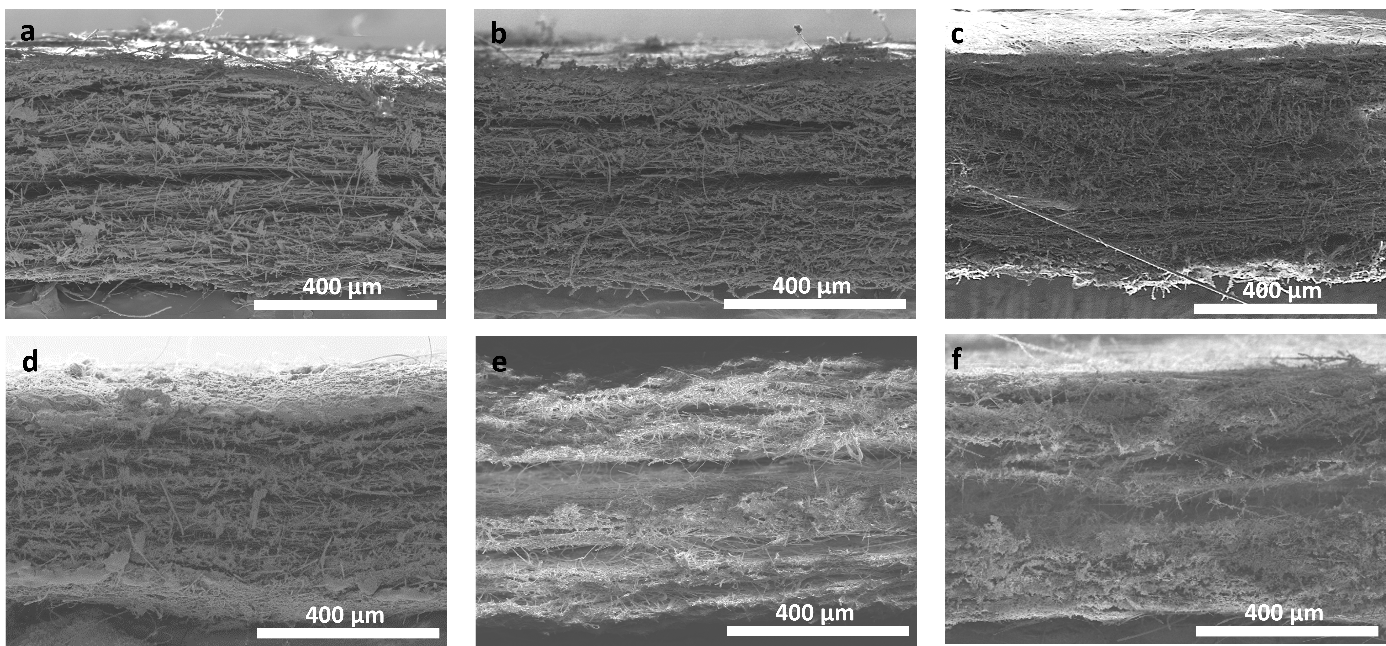


**Fig. S24** **a-f,** Cross-sectional SEM images of pristine separators: GHF@ZIF-8 (**a**), GHF@HKUST-1 (**b**) and GHF@ZIF-8@HKUST-1 (**c**); and the post-cycled separators: GHF@ZIF-8 (**d**), GHF@HKUST-1 (**e**) and GHF@ZIF-8@HKUST-1 (**f**)

**Note S18 The rate capability of Li//LFP cells using core-shell MOF-based QSSE**

In addition, the rate capability of Li//LFP cells employing the GHF@ZIF-8@HKUST-1 QSSE was systematically evaluated at 100 °C under different current densities (0.1 C, 0.3 C, 0.5 C, 1 C, and 2 C) (Fig. S25). The cell delivered an initial discharge capacity of ~165 mAh g⁻¹ at 0.1 C, gradually decreasing to ~160 mAh g⁻¹ at 0.3 C, ~155 mAh g⁻¹ at 0.5 C, ~150 mAh g⁻¹ at 1 C, and ~130 mAh g⁻¹ at 2 C. When the current density was returned to 0.1 C, the capacity nearly recovered to its initial value, demonstrating the reversibility and structural stability of the MOF-based electrolyte even under harsh high-temperature and high-rate conditions. Coulombic efficiency remained close to 97–100% throughout the tests, further confirming stable Li⁺ transport without parasitic side reactions.


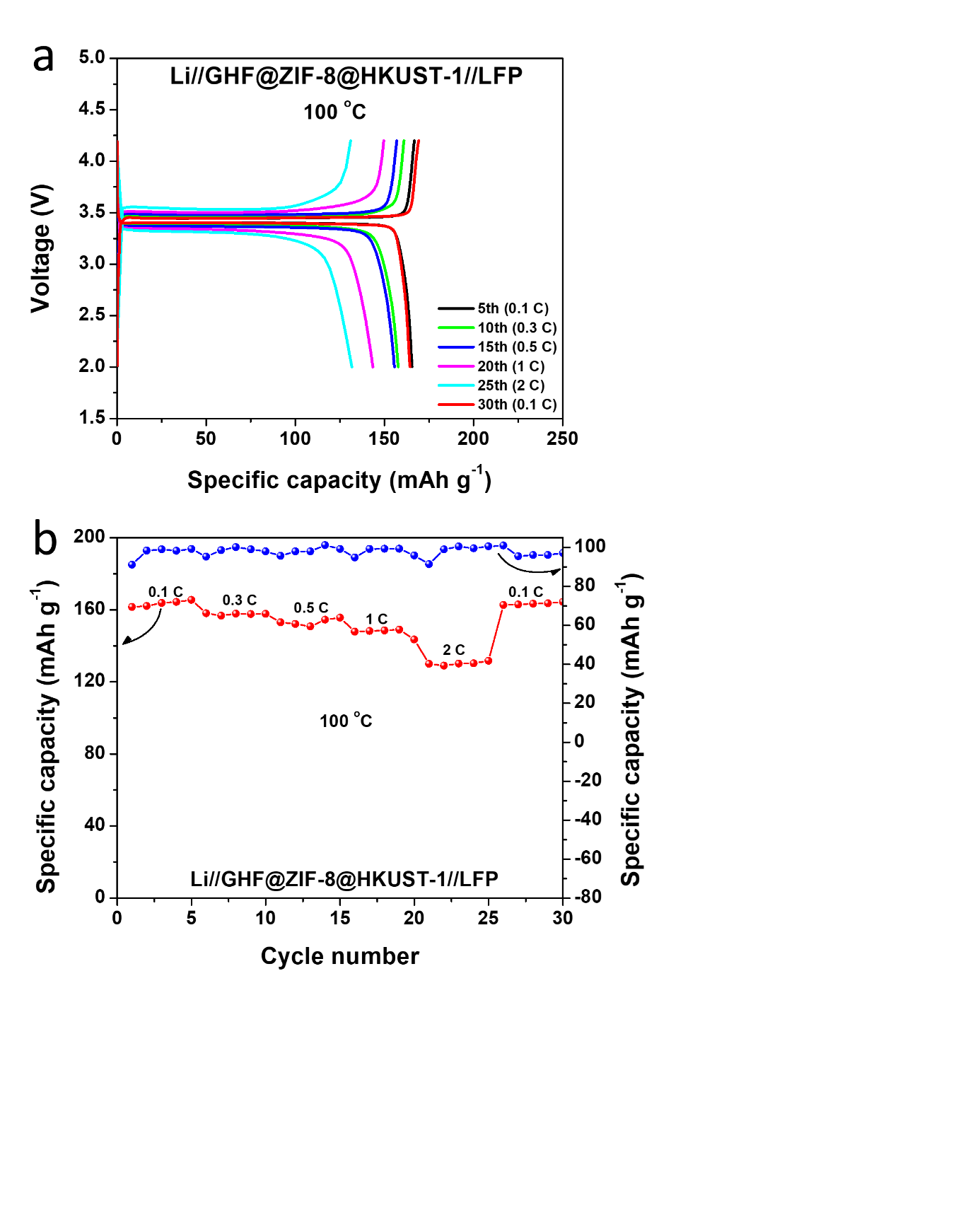


**Fig. S25 a, b**, The cyclic performance of Li//GHF@ZIF-8@HKUST-1//LFP at 100 ^o^C with various C rates (0.1, 0.3, 0.5, 1, and 2 C)

**Note S19 The initial approach of large-scale pouch cell designed by 1D core-shell MOF-based QSSE at 100 ^o^C**

To demonstrate the scalability and practical applicability of our QSSE design, a full pouch cell (**Fig. S**26) was assembled using a graphite anode and a high-loading LiFePO_4_ (LFP) cathode with an active surface area of 13.44 cm². The anode was prepared by mixing graphite powder, Super P, and PVDF binder in a mass ratio of 8:1:1 using N-methyl-2-pyrrolidone (NMP) as the solvent. The resulting slurry was uniformly cast onto copper foil via a blade-casting method and dried under vacuum at 110 °C for 12 hours prior to pouch cell assembly. The areal loading of active material was 3.2 mg cm⁻² for the cathode and 3.58 mg cm⁻² for the anode. The pouch cell was tested for one charge–discharge cycle at a rate of 0.1 C. As shown in the galvanostatic charge–discharge (GCD) profile, the initial discharge capacity reached 133.06 mAh g^-1^ (Fig. S26). After calculation, the total output capacity of the pouch cell was 6.8 mAh, which is significantly higher than that of a coin cell using the same LFP cathode (0.78 mAh). This substantial increase in output current over time highlights the excellent scalability of our QSSE architecture and its strong potential for real-world energy storage applications. These promising results suggest that our QSSE design could serve as a viable platform for the future development of high-performance lithium metal batteries at practical device scales.

**Fig. S26 a**, Digital photo of prepared pouch cell using the designed MOF-based QSSE. **b**, The initial galvanostatic charge-discharge (GCD) curve of pouch cell at 0.1 C and very high temperature (100 ^o^C)

**Supplementary References**

1. M.H. Nguyen, N.M. Ngo, B.-K. Kim, S. Park, Dual ionic pathways in semi-solid electrolyte based on binary metal–organic frameworks enable stable operation of Li-metal batteries at extremely high temperatures. Adv. Sci. **11**(43), 2407018 (2024). <https://doi.org/10.1002/advs.202407018>
2. X.-H. Shi, X.-L. Li, Y.-M. Li, Z. Li, D.-Y. Wang, Flame-retardant strategy and mechanism of fiber reinforced polymeric composite: a review. Compos. Part B Eng. **233**, 109663 (2022). <https://doi.org/10.1016/j.compositesb.2022.109663>
3. W. Liu, Y. Zhu, C. Qian, H. Dai, Y. Fu et al., Interfacial modification between glass fiber and polypropylene using a novel waterborne amphiphilic sizing agent. Compos. Part B Eng. **241**, 110029 (2022). <https://doi.org/10.1016/j.compositesb.2022.110029>
4. Y. Wu, Y. Song, D. Wu, X. Mao, X. Yang et al., Recent progress in modifications, properties, and practical applications of glass fiber. Molecules **28**(6), 2466 (2023). <https://doi.org/10.3390/molecules28062466>
5. P. Newsholme, L. Stenson, M. Sulvucci, R. Sumayao, M. Krause, Amino acid metabolism. In: Comprehensive Biotechnology, pp. 3–14. Elsevier (2011). <https://doi.org/10.1016/b978-0-08-088504-9.00002-7>
6. T.D. Tavares, J.C. Antunes, F. Ferreira, H.P. Felgueiras, Biofunctionalization of natural fiber-reinforced biocomposites for biomedical applications. Biomolecules **10**(1), 148 (2020). <https://doi.org/10.3390/biom10010148>
7. N.D. Tissera, R.N. Wijesena, H. Yasasri, K.M.N. de Silva, R.M. de Silva, Fibrous keratin protein bio micro structure for efficient removal of hazardous dye waste from water: Surface charge mediated interfaces for multiple adsorption desorption cycles. Mater. Chem. Phys. **246**, 122790 (2020). <https://doi.org/10.1016/j.matchemphys.2020.122790>
8. A. Yıldız, A.A. Kara, F. Acartürk, Peptide-protein based nanofibers in pharmaceutical and biomedical applications. Int. J. Biol. Macromol. **148**, 1084–1097 (2020). <https://doi.org/10.1016/j.ijbiomac.2019.12.275>
9. S.S. Kunde, S. Wairkar, Targeted delivery of albumin nanoparticles for breast cancer: a review. Colloids Surf. B Biointerfaces **213**, 112422 (2022). <https://doi.org/10.1016/j.colsurfb.2022.112422>
10. R.R. Kudarha, K.K. Sawant, Albumin based versatile multifunctional nanocarriers for cancer therapy: Fabrication, surface modification, multimodal therapeutics and imaging approaches. Mater. Sci. Eng. C **81**, 607–626 (2017). <https://doi.org/10.1016/j.msec.2017.08.004>
11. A.O. Elzoghby, W.M. Samy, N.A. Elgindy, Albumin-based nanoparticles as potential controlled release drug delivery systems. J. Control. Release **157**(2), 168–182 (2012). <https://doi.org/10.1016/j.jconrel.2011.07.031>
12. A.-G. Nguyen, R. Verma, G.-C. Song, J. Kim, C.-J. Park, *In situ* polymerization on a 3D ceramic framework of composite solid electrolytes for room-temperature solid-state batteries. Adv. Sci. **10**(21), 2207744 (2023). <https://doi.org/10.1002/advs.202207744>
13. S.A.S. Mohammed, W.Z.N. Yahya, M.A. Bustam, M.G. Kibria, Experimental and computational evaluation of 1, 2, 4-triazolium-based ionic liquids for carbon dioxide capture. Separations **10**(3), 192 (2023). <https://doi.org/10.3390/separations10030192>
14. Y. Zhu, Q. Liu, J. Caro, A. Huang, Highly hydrogen-permselective zeolitic imidazolate framework ZIF-8 membranes prepared on coarse and macroporous tubes through repeated synthesis. Sep. Purif. Technol. **146**, 68–74 (2015). <https://doi.org/10.1016/j.seppur.2015.03.020>
15. Y. Mao, B. Su, W. Cao, J. Li, Y. Ying et al., Specific oriented metal-organic framework membranes and their facet-tuned separation performance. ACS Appl. Mater. Interfaces **6**(18), 15676–15685 (2014). <https://doi.org/10.1021/am5049702>
16. M. Pathak, A. Mahun, P. Černoch, Z. Morávková, Swelling mechanism of polyoxazoline-based gel polymer electrolytes for lithium-ion batteries. ACS Appl. Polym. Mater. **7**(4), 2371–2383 (2025). <https://doi.org/10.1021/acsapm.4c03473>
17. G.J. Janz, J. Ambrose, J.W. Coutts, J.R. Downey, Raman spectrum of propylene carbonate. Spectrochim. Acta Part A Mol. Spectrosc. **35**(2), 175–179 (1979). <https://doi.org/10.1016/0584-8539(79)80181-6>
18. S. Takamoto, C. Shinagawa, D. Motoki, K. Nakago, W. Li et al., Towards universal neural network potential for material discovery applicable to arbitrary combination of 45 elements. Nat. Commun. **13**, 2991 (2022). <https://doi.org/10.1038/s41467-022-30687-9>
19. K. Hisama, G. Valadez Huerta, M. Koyama, Molecular dynamics of liquid–electrode interface by integrating Coulomb interaction into universal neural network potential. J. Comput. Chem. **45**(32), 2805–2811 (2024). <https://doi.org/10.1002/jcc.27487>
20. M.S. Daw, M.I. Baskes, Embedded-atom method: Derivation and application to impurities, surfaces, and other defects in metals. Phys. Rev. B **29**(12), 6443–6453 (1984). <https://doi.org/10.1103/physrevb.29.6443>
21. J. Tersoff, New empirical model for the structural properties of silicon. Phys. Rev. Lett. **56**(6), 632–635 (1986). <https://doi.org/10.1103/PhysRevLett.56.632>
22. A.C.T. van Duin, S. Dasgupta, F. Lorant, W.A. Goddard, ReaxFF:   a reactive force field for hydrocarbons. J. Phys. Chem. A **105**(41), 9396–9409 (2001). <https://doi.org/10.1021/jp004368u>
23. S. Choung, W. Park, J. Moon, J.W. Han, Rise of machine learning potentials in heterogeneous catalysis: Developments, applications, and prospects. Chem. Eng. J. **494**, 152757 (2024). <https://doi.org/10.1016/j.cej.2024.152757>
24. A. Martínez-Juárez, C. Pecharromán, J.E. Iglesias, J.M. Rojo, Relationship between activation energy and bottleneck size for Li^+^ ion conduction in NASICON materials of composition LiMM‘(PO4)_3_; M, M‘= Ge, Ti, Sn, Hf. J. Phys. Chem. B **102**(2), 372–375 (1998). <https://doi.org/10.1021/jp973296c>
